# Novel botanical active component derivatives containing carboxamide and 1,3,4-Thiadiazole thioether moieties: Design, synthesis, and inhibitory activity

**DOI:** 10.3389/fchem.2022.1036909

**Published:** 2022-09-27

**Authors:** Pei Li, Cong Chen, Rongxi Zhu, Guixia Yang, Min Xu, Guanghua Wan, Xiang Wang

**Affiliations:** Qiandongnan Engineering and Technology Research Center for Comprehensive Utilization of National Medicine, Kaili University, Kaili, China

**Keywords:** botanical active component, carboxamide, 1,3,4-thiadiazole thioether, antibacterial activity, antifungal activity

## Abstract

In this study, using the botanical active components of carvacrol, thymol, guaiacol, and sesamol as the lead structures, 19 novel botanical active component derivatives containing carboxamide and 1,3,4-thiadiazole thioether moieties (**5a**−**5s**) were synthesized and structurally characterized by ^1^H NMR, ^13^C NMR, and HRMS. The antibacterial bioassay results *in vitro* showed that compound 2-(2-methoxyphenoxy)-N-(5-(methylthio)-1,3,4-thiadiazol-2-yl)acetamide (**5k**) revealed excellent inhibitory activities against Xanthomonas axonopodis pv. citri (Xac) and Xanthomonas oryzae pv. oryzicolaby (Xoc), with the median effective concentration (EC_50_) values of 22 and 15 μg/ml, respectively, which were even better than those of thiodiazole copper and bismerthiazol. Meanwhile, all the target compounds revealed lower *in vitro* inhibitory effects on Mucor bainieri (M. bainieri), Mucor fragilis (M. fragilis), and Trichoderma atroviride (T. atroviride), than carbendazim.

## 1 Introduction

As a serious threat to agricultural production, plant diseases can cause huge economic losses every year (Rosegrant and Cline, 2003; Neeraja et al., 2010; Opara, 2013; Bhattacharjee and Dey, 2014). Although the use of pesticides is an effective method to control plant diseases, the frequent use of traditional pesticides can lead to many negative effects such as pathogenic microorganism resistance, environmental contamination, and human health (Guo et al., 1998). As the improving of human living level and the demand for high-quality agricultural products, a limit on the use of traditional pesticides is required (Chávez-Dulanto et al., 2021).

**FIGURE 1:**
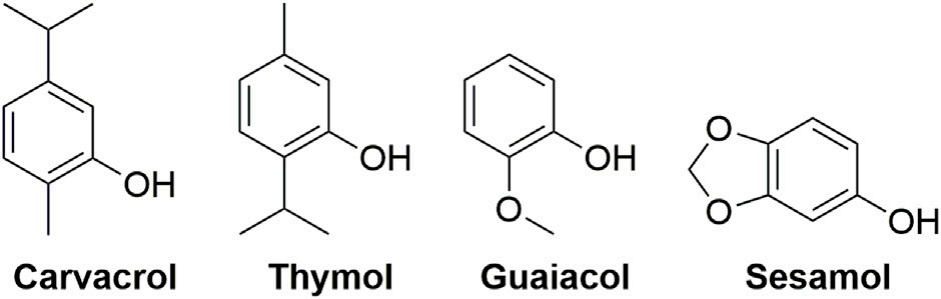
The structures of botanical active components of carvacrol, thymol, guaiacol, and sesamol.

**FIGURE 2:**
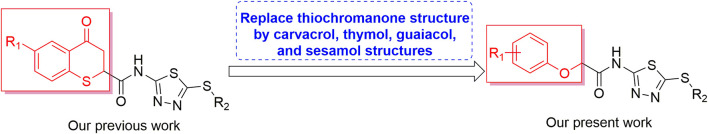
Design route of the target compounds 5a−5s.

In the 21st century and beyond, use of natural product pesticides to control plant diseases is an innovative approach of sustainable agricultural development (Cantrell et al., 2020; Souto et al., 2021). It is a critical approach to find new active components and to develop new pesticides by modifying the structure of natural products. Botanical active components of carvacrol, thymol, guaiacol, and sesamol (Figure 1) had a broad spectrum of pesticide biological properties, such as antifungal and insecticidal activity (Shen and He, 2022; Cui et al., 2022; Jia et al., 2007; Sharifi-Rad et al., 2018; Karina Kachur, 2020; Rathod et al., 2021). However, the inhibitory effects on plant pathogenic bacteria diseases of carvacrol, thymol, guaiacol, sesamol and their derivative had not been reported yet. Meanwhile, the carboxamide and 1,3,4-thiadiazole thioether moieties had extensive pesticide biological activities, including antibacterial, antifungal, antiviral, and insecticidal activity (Dalgaard, et al., 1994; Wu et al., 2016; Yang et al., 2018; Chen, et al., 2019; Yang et al., 2019; Tang et al., 2020; Chen et al., 2021). In our previous work, a series of novel thiochromanone derivatives containing carboxamide and 1,3,4-thiadiazole thioether moieties (Figure 2) were prepared and demonstrated to have suitable antibacterial and antifungal activity (Yu et al., 2020).

To develop new lead compounds, in this study, we aimed to replace thiochromanone structure in the structure of our reported structures by carvacrol, thymol, guaiacol, and sesamol structures to build some new botanical active component derivatives containing carboxamide and 1,3,4-thiadiazole thioether moieties (Figure 2).

## 2 Materials and methods

### 2.1 Chemical synthesis

#### 2.1.1 Preparation of intermediates 2 and 4

**Scheme 1:**
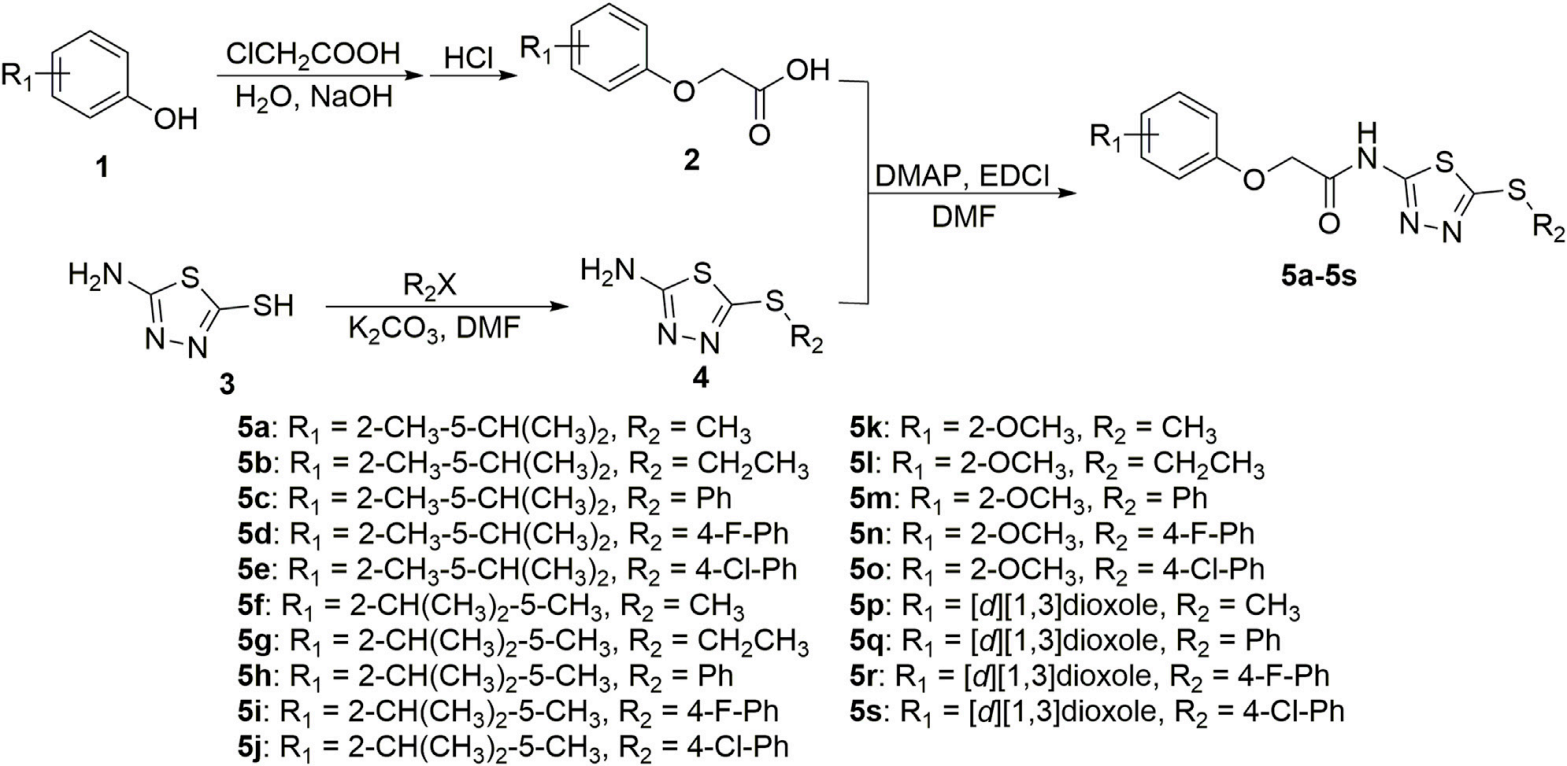
Synthetic route for compounds 5a−5s.

As shown in Scheme 1, using the botanical active components of carvacrol, thymol, guaiacol, and sesamol as the lead structures, intermediates 2 and 4 were prepared using the methods that have been previously reported (Friedrich et al., 2020; Yu et al., 2022).

#### 2.1.2 Preparation of the target compounds 5a−5s

To a 25 ml round bottom flask, intermediates 2 (20 mmol) and 4 (20 mmol) dissolved in DMF (10 ml), DMAP (2 mmol), and EDCI (30 mmol) were added. After reacting overnight at room temperature, the precipitates obtained by adding distilled water (50 ml) were recrystallized from ethyl acetate to give the target compounds 5a−5s.

### 2.2 Bioactivity evaluation

The preliminary inhibitory effects results *in vitro* of compounds 5a−5s against Xanthomonas axonopodis pv. citri (Xac) and Xanthomonas oryzae pv. oryzicolaby (Xoc) as well as Mucor bainieri (M. bainieri), Mucor fragilis (M. fragilis), and Trichoderma atroviride (T. atroviride) were determined by the turbidimeter test (for antibacterial activity test) and mycelial growth rate method (for antifungal activity test) (Schaad et al., 1996; Wang et al., 2022). Meanwhile, the median effective concentration (EC_50_) values of compounds 5a, 5b, 5f, 5k, 5L, and 5n against Xac and Xoc were calculated using the SPSS 19.0 software (SPSS, Chicago, United States).

## 3 Results and discussion

### 3.1 Chemistry

Using the botanical active components of carvacrol, thymol, guaiacol, and sesamol as the lead structures, compounds 5a−5s were prepared in three steps, namely, substitution, thioetherification, and condensation reaction, with the yields of 68%–88% and the melting point ranges within two degrees centigrades. In the ^1^H NMR spectra of compounds 5a−5s, a singlet at 12.87–12.79 and 4.93–4.81 ppm indicated H atom in CONH and OCH_2_ groups, respectively. Meanwhile, a singlet at 168.07–167.79 ppm in the ^13^C NMR spectra indicated C atom in CONH group. In addition, the molecular weights of compounds 5a−5s were assigned by combining the [M + Na]^+^ ions with the confidence level of 100%. The physical and chemical properties and spectra data for compounds 5a−5s are presented in the following.

2-(5-Isopropyl-2-methylphenoxy)-N-(5-(methylthio)-1,3,4-thiadiazol-2-yl)acetamide (5a). White solid, yield 77%, mp 152–154°C; ^1^H NMR (400 MHz, DMSO-d_6_) *δ* (ppm): 12.85 (s, 1H), 7.06 (d, J = 8.0 Hz, 1H), 6.75 (d, J = 8.0 Hz, 1H), 6.72 (s, 1H), 4.93 (s, 2H), 2.83–2.76 (m, 1H), 2.72 (s, 3H), 2.99 (s, 3H), 1.15 (s, 3H), 1.13 (s, 3H); ^13^C NMR (100 MHz, DMSO-d_6_) *δ* (ppm): 167.99, 161.19, 158.17, 156.19, 147.85, 130.90, 123.95, 119.18, 110.14, 66.82, 33.77, 24.35, 16.38, 16.14; Anal. calcd. for m/z of C_15_H_19_N_3_O_2_S_2_ (HRMS [M + Na]^+^): 360.08109, found: 360.08046.

N-(5-(ethylthio)-1,3,4-thiadiazol-2-yl)-2-(5-isopropyl-2-methylphenoxy) acetamide (5b). White solid, yield 74%, mp 130–131°C; ^1^H NMR (400 MHz, DMSO-d_6_) *δ* (ppm): 12.86 (s, 1H), 7.06 (d, J = 8.0 Hz, 1H), 6.75 (d, J = 8.0 Hz, 1H), 6.71 (s, 1H), 4.93 (s, 2H), 3.23 (q, J1 = 8.0 Hz, J2 = 16.0 Hz, 2H), 2.83–2.76 (m, 1H), 2.18 (s, 3H), 1.34 (t, J = 8.0 Hz, 3H), 1.15 (s, 3H), 1.13 (s, 3H); ^13^C NMR (100 MHz, DMSO-d_6_) *δ* (ppm): 168.04, 159.49, 158.53, 156.18, 147.86, 130.91, 123.94, 119.18, 110.15, 66.82, 33.76, 28.52, 24.35, 16.15, 15.17; Anal. calcd. for m/z of C_16_H_21_N_3_O_2_S_2_ (HRMS [M + Na]^+^): 374.09674, found: 374.09643.

N-(5-(benzylthio)-1,3,4-thiadiazol-2-yl)-2-(5-isopropyl-2-methylphenoxy) acetamide (5c). White solid, yield 81%, mp 137–138°C; ^1^H NMR (400 MHz, DMSO-d_6_) *δ* (ppm): 12.86 (s, 1H), 7.40 (d, J = 8.0 Hz, 2H), 7.34–7.25 (m, 3H), 7.06 (d, J = 8.0 Hz, 1H), 6.75 (d, J = 8.0 Hz, 1H), 6.69 (s, 1H), 4.92 (s, 2H), 4.49 (s, 2H), 2.83–2.76 (m, 1H), 2.17 (s, 3H), 1.14 (s, 3H), 1.13 (s, 3H); ^13^C NMR (100 MHz, DMSO-d_6_) *δ* (ppm): 168.06, 158.90, 156.16, 147.85, 137.14, 130.91, 129.46, 129.01, 128.06, 123.91, 119.17, 110.11, 66.78, 38.02, 33.76, 24.35, 16.15; Anal. calcd. for m/z of C_21_H_23_N_3_O_2_S_2_ (HRMS [M + Na]^+^): 436.11239, found: 436.11185.

N-(5-((4-fluorobenzyl)thio)-1,3,4-thiadiazol-2-yl)-2-(5-isopropyl-2-methylphenoxy)acetamide (5d). Yellow solid, yield 86%, mp 140–141°C; ^1^H NMR (400 MHz, DMSO-d_6_) *δ* (ppm): 12.86 (s, 1H), 7.47–7.43 (m, 2H), 7.16 (d, J = 8.0 Hz, 2H), 7.05 (s, 1H), 6.75 (d, J = 8.0 Hz, 1H), 6.70 (s, 1H), 4.93 (s, 2H), 4.49 (s, 2H), 2.83–2.76 (m, 1H), 2.18 (s, 3H), 1.14 (s, 3H), 1.13 (s, 3H); ^13^C NMR (100 MHz, DMSO-d_6_) *δ* (ppm): 168.07, 161.98 (d, J = 243.0 Hz), 158.69, 158.16, 147.85, 133.50 (d, J = 3.0 Hz), 131.51 (d, J = 9.0 Hz), 130.91, 123.92, 119.18, 115.90, 115.69, 110.12, 66.80, 37.14, 33.76, 24.34, 16.14; Anal. calcd. for m/z of C_21_H_22_FN_3_O_2_S_2_ (HRMS [M + Na]^+^): 454.10297, found: 454.10241.

N-(5-((4-chlorobenzyl)thio)-1,3,4-thiadiazol-2-yl)-2-(5-isopropyl-2-methylphenoxy)acetamide (**5e**). Yellow solid, yield 74%, mp 132–134°C; ^1^H NMR (400 MHz, DMSO-d_6_) *δ* (ppm): 12.86 (s, 1H), 7.44–7.37 (m, 1H), 7.06 (d, J = 8.0 Hz, 1H), 6.75 (d, J = 8.0 Hz, 1H), 6.70 (s, 1H), 4.93 (s, 2H), 4.49 (s, 2H), 2.83–2.76 (m, 1H), 2.18 (s, 3H), 1.14 (s, 3H), 1.13 (s, 3H); ^13^C NMR (100 MHz, DMSO-d_6_) *δ* (ppm): 168.06, 158.97, 158.56, 156.16, 147.85, 136.45, 132.64, 131.30, 130.91, 128.94, 123.92, 119.18, 110.13, 66.80, 37.14, 33.76, 24.34, 16.14; Anal. calcd. for m/z of C_21_H_22_ClN_3_O_2_S_2_ (HRMS [M + Na]^+^): 470.07342, found: 470.07318.

2-(2-Isopropyl-5-methylphenoxy)-N-(5-(methylthio)-1,3,4-thiadiazol-2-yl)acetamide (**5f**). White solid, yield 79%, mp 158–160 °C; ^1^H NMR (400 MHz, DMSO-d_6_) *δ* (ppm): 12.84 (s, 1H), 7.09 (d, J = 8.0 Hz, 1H), 6.75 (d, J = 8.0 Hz, 1H), 6.66 (s, 1H), 4.91 (s, 2H), 2.72 (s, 3H), 2.23 (s, 3H), 1.17 (s, 3H), 1.16 (s, 3H); ^13^C NMR (100 MHz, DMSO-d_6_) *δ* (ppm): 167.83, 161.15, 158.21, 155.19, 136.33, 126.31, 122.32, 112.82, 66.73, 26.42, 23.15, 21.40, 16.43; Anal. calcd. for m/z of C_15_H_19_N_3_O_2_S_2_ (HRMS [M + Na]^+^): 360.08109, found: 360.08075.

N-(5-(ethylthio)-1,3,4-thiadiazol-2-yl)-2-(2-isopropyl-5-methylphenoxy)acetamide (5g). White solid, yield 78%, mp 168–170°C; ^1^H NMR (400 MHz, DMSO-d_6_) *δ* (ppm): 12.87 (s, 1H), 7.09 (d, J = 8.0 Hz, 1H), 6.75 (d, J = 8.0 Hz, 1H), 6.67 (s, 1H), 4.91 (s, 2H), 3.34–3.29 (m, 1H), 3.23 (q, J1 = 8.0 Hz, J2 = 16.0 Hz, 2H), 2.23 (s, 3H), 1.35 (t, J = 8.0 Hz, 3H), 1.17 (s, 3H), 1.16 (s, 3H); ^13^C NMR (100 MHz, DMSO-d_6_) *δ* (ppm): 167.88, 159.45, 158.57, 155.18, 136.34, 133.91, 126.31, 122.33, 112.80, 66.72, 28.54, 26.42, 23.15, 21.40, 15.20; Anal. calcd. for m/z of C_16_H_21_N_3_O_2_S_2_ (HRMS [M + Na]^+^): 374.09674, found: 374.09632.

N-(5-(benzylthio)-1,3,4-thiadiazol-2-yl)-2-(2-isopropyl-5-methylphenoxy)acetamide (**5h**). White solid, yield 88%, mp 133–135°C; ^1^H NMR (400 MHz, DMSO-d_6_) *δ* (ppm): 12.86 (s, 1H), 7.41 (d, J = 8.0 Hz, 2H), 7.35–7.25 (m, 3H), 7.09 (d, J = 8.0 Hz, 1H), 6.75 (d, J = 8.0 Hz, 1H), 6.66 (s, 1H), 4.91 (s, 2H), 4.50 (s, 2H), 3.34–3.27 (m, 1H), 2.23 (s, 3H), 1.17 (s, 3H), 1.15 (s, 3H); ^13^C NMR (100 MHz, DMSO-d_6_) *δ* (ppm): 167.89, 158.88, 155.17, 137.13, 136.33, 133.91, 129.45, 129.02, 128.06, 126.30, 122.34, 112.82, 66.74, 38.01, 26.40, 23.16, 21.40; Anal. calcd. for m/z of C_21_H_23_N_3_O_2_S_2_ (HRMS [M + Na]^+^): 436.11239, found: 436.11185.

N-(5-((4-fluorobenzyl)thio)-1,3,4-thiadiazol-2-yl)-2-(2-isopropyl-5-methylphenoxy)acetamide (**5i**). White solid, yield 78%, mp 129–130°C; ^1^H NMR (400 MHz, DMSO-d_6_) *δ* (ppm): 12.86 (s, 1H), 7.46 (q, J1 = 4.0 Hz, J2 = 8.0 Hz, 2H), 7.16 (t, J = 8.0 Hz, 2H), 7.09 (d, J = 8.0 Hz, 1H), 6.75 (d, J = 8.0 Hz, 1H), 6.66 (s, 1H), 4.91 (s, 2H), 4.49 (s, 2H), 3.34–3.27 (m, 1H), 2.23 (s, 3H), 1.17 (s, 3H), 1.15 (s, 3H); ^13^C NMR (100 MHz, DMSO-d_6_) *δ* (ppm): 167.91, 161.98 (d, J = 242.0 Hz), 158.98, 158.65, 155.17, 136.33, 133.91, 133.52 (d, J = 3.0 Hz), 131.51 (d, J = 8.0 Hz), 126.31, 122.34, 115.82 (d, J = 21.0 Hz), 112.82, 66.74, 37.13, 26.39, 23.15, 21.39; Anal. calcd. for m/z of C_21_H_22_FN_3_O_2_S_2_ (HRMS [M + Na]^+^): 454.10297, found: 454.10236.

N-(5-((4-chlorobenzyl)thio)-1,3,4-thiadiazol-2-yl)-2-(2-isopropyl-5-methylphenoxy)acetamide (5j). White solid, yield 82%, mp 138–140°C; ^1^H NMR (400 MHz, DMSO-d_6_) *δ* (ppm): 12.85 (s, 1H), 7.41 (q, J1 = 8.0 Hz, J2 = 16.0 Hz, 4H), 7.09 (d, J = 8.0 Hz, 1H), 6.75 (d, J = 8.0 Hz, 1H), 6.65 (s, 1H), 4.90 (s, 2H), 4.49 (s, 2H), 3.31–3.26 (m, 1H), 2.23 (s, 3H), 1.17 (s, 3H), 1.15 (s, 3H); ^13^C NMR (100 MHz, DMSO-d_6_) *δ* (ppm): 167.83, 158.53, 155.16, 136.49, 136.34, 133.91, 132.63, 131.31, 128.97, 126.32, 122.34, 112.82, 66.72, 37.11, 26.39, 23.16, 21.40; Anal. calcd. for m/z of C_21_H_22_ClN_3_O_2_S_2_ (HRMS [M + Na]^+^): 470.07342, found: 470.07319.

2-(2-Methoxyphenoxy)-N-(5-(methylthio)-1,3,4-thiadiazol-2-yl)acetamide (**5k**). Yellow solid, yield 72%, mp 135–136 °C; ^1^H NMR (400 MHz, DMSO-d_6_) *δ* (ppm): 12.79 (s, 1H), 7.02 (d, J = 4.0 Hz, 1H), 6.95 (q, J1 = 8.0 Hz, J2 = 16.0 Hz, 2H), 6.86 (t, J = 8.0 Hz, 1H), 4.88 (s, 2H), 3.79 (s, 3H), 2.72 (s, 3H); ^13^C NMR (100 MHz, DMSO-d_6_) *δ* (ppm): 167.82, 161.17, 158.14, 149.66, 147.64, 122.64, 121.12, 114.87, 113.03, 67.61, 56.03, 16.42; Anal. calcd. for m/z of C_12_H_13_N_3_O_3_S_2_ (HRMS [M + Na]^+^): 334.02905, found: 334.02896.

N-(5-(ethylthio)-1,3,4-thiadiazol-2-yl)-2-(2-methoxyphenoxy)acetamide (5l). White solid, yield 68%, mp 138–140°C; ^1^H NMR (400 MHz, DMSO-d_6_) *δ* (ppm): 12.81 (s, 1H), 7.02 (dd, J1 = 4.0 Hz, J2 = 8.0 Hz, 1H), 6.95 (qd, J1 = 4.0 Hz, J2 = 8.0 Hz, 2H), 6.89–6.84 (m, 1H), 4.89 (s, 2H), 3.79 (s, 3H), 3.23 (q, J1 = 8.0 Hz, J2 = 16.0 Hz, 2H), 1.34 (t, J = 8.0 Hz, 3H); ^13^C NMR (100 MHz, DMSO-d_6_) *δ* (ppm): 167.88, 159.45, 158.51, 149.65, 147.64, 122.63, 121.12, 114.84, 113.02, 67.59, 56.03, 28.54, 15.19; Anal. calcd. for m/z of C_13_H_15_N_3_O_3_S_2_ (HRMS [M + Na]^+^): 348.04470, found: 348.04468.

N-(5-(benzylthio)-1,3,4-thiadiazol-2-yl)-2-(2-methoxyphenoxy)acetamide (5m). White solid, yield 85%, mp 135–136°C; ^1^H NMR (400 MHz, DMSO-d_6_) *δ* (ppm): 12.80 (s, 1H), 7.41 (d, J = 8.0 Hz, 2H), 7.35–7.25 (m, 3H), 7.02–6.84 (m, 4H), 4.87 (s, 2H), 4.49 (s, 2H), 3.78 (s, 3H); ^13^C NMR (100 MHz, DMSO-d_6_) *δ* (ppm): 167.87, 158.89, 158.71, 149.63, 147.61, 137.14, 129.47, 129.03, 128.07, 122.63, 121.11, 114.80, 113.00, 67.55, 56.01, 37.99; Anal. calcd. for m/z of C_18_H_17_N_3_O_3_S_2_ (HRMS [M + Na]^+^): 410.06035, found: 410.06027.

N-(5-((4-fluorobenzyl)thio)-1,3,4-thiadiazol-2-yl)-2-(2-methoxyphenoxy)acetamide (5n). Yellow solid, yield 77%, mp 137–139°C; ^1^H NMR (400 MHz, DMSO-d_6_) *δ* (ppm): 12.81 (s, 1H), 7.47–7.43 (m, 2H), 7.19–7.14 (m, 2H), 7.03–6.84 (m, 4H), 4.88 (s, 2H), 4.49 (s, 2H), 3.79 (s, 3H); ^13^C NMR (100 MHz, DMSO-d_6_) *δ* (ppm): 167.90, 161.99 (d, J = 243.0 Hz), 158.93, 158.66, 149.65, 147.62, 133.53 (d, J = 3.0 Hz), 131.54 (d, J = 8.0 Hz), 122.64, 121.11, 115.82 (d, J = 22.0 Hz), 114.84, 113.01, 67.59, 56.02, 37.13; Anal. calcd. for m/z of C_18_H_16_FN_3_O_3_S_2_ (HRMS [M + Na]^+^): 428.05093, found: 428.05066.

N-(5-((4-chlorobenzyl)thio)-1,3,4-thiadiazol-2-yl)-2-(2-methoxyphenoxy)acetamide (**5o**). Yellow solid, yield 79%, mp 136–138 °C; ^1^H NMR (400 MHz, DMSO-d_6_) *δ* (ppm): 12.81 (s, 1H), 7.44–7.38 (m, 4H), 7.03–6.84 (m, 4H), 4.88 (s, 2H), 4.49 (s, 2H), 3.79 (s, 3H); ^13^C NMR (100 MHz, DMSO-d_6_) *δ* (ppm): 167.91, 158.97, 158.54, 149.65, 147.62, 136.49, 132.64, 131.32, 128.97, 122.64, 121.12, 114.85, 113.01; 67.59, 56.02, 37.13; Anal. calcd. for m/z of C_18_H_16_ClN_3_O_3_S_2_ (HRMS [M + Na]^+^): 444.02138, found: 444.02089.

2-(Benzo[d][1,3]dioxol-5-yloxy)-N-(5-(methylthio)-1,3,4-thiadiazol-2-yl)acetamide (5p). Pink solid, yield 78%, mp 171–172°C; ^1^H NMR (400 MHz, DMSO-d_6_) *δ* (ppm): 12.85 (s, 1H), 6.82 (d, J = 8.0 Hz, 1H), 6.70 (s, 1H), 6.40 (d, J = 8.0 Hz, 1H), 5.97 (s, 2H), 4.82 (s, 2H), 2.72 (s, 3H); ^13^C NMR (100 MHz, DMSO-d_6_) *δ* (ppm): 167.79, 161.19, 158.16, 153.47, 148.37, 142.21, 108.43, 106.36, 101.63, 98.61, 67.38, 16.41; Anal. calcd. for m/z of C_12_H_11_N_3_O_4_S_2_ (HRMS [M + Na]^+^): 348.00832, found: 348.00793.

2-(Benzo[d][1,3]dioxol-5-yloxy)-N-(5-(benzylthio)-1,3,4-thiadiazol-2-yl)acetamide (5q). Pink solid, yield 84%, mp 176–178°C; ^1^H NMR (400 MHz, DMSO-d_6_) *δ* (ppm): 12.82 (s, 1H), 7.41 (d, J = 8.0 Hz, 2H), 7.35–7.26 (m, 3H), 6.81 (d, J = 8.0 Hz, 1H), 6.70 (d, J = 4.0 Hz, 1H), 6.40 (s, 1H), 5.97 (s, 2H), 4.81 (s, 2H), 4.49 (s, 2H); ^13^C NMR (100 MHz, DMSO-d_6_) *δ* (ppm): 167.86, 158.93, 153.46, 148.38, 142.22, 137.13, 129.46, 129.03, 128.07, 108.43, 106.34, 101.64, 98.60, 67.36, 38.01; Anal. calcd. for m/z of C_18_H_15_N_3_O_4_S_2_ (HRMS [M + Na]^+^): 424.03962, found: 424.03902.

2-(Benzo[d][1,3]dioxol-5-yloxy)-N-(5-((4-fluorobenzyl)thio)-1,3,4-thiadiazol-2-yl)acetamide (**5r**). Yellow solid, yield 79%, mp 165–167°C; ^1^H NMR (400 MHz, DMSO-d_6_) *δ* (ppm): 12.83 (s, 1H), 7.47–7.44 (m, 2H), 7.17 (d, J = 8.0 Hz, 2H), 6.82 (d, J = 8.0 Hz, 1H), 6.40 (q, J1 = 4.0 Hz, J2 = 8.0 Hz, 1H), 5.97 (s, 2H), 4.82 (s, 2H), 4.49 (s, 2H); ^13^C NMR (100 MHz, DMSO-d_6_) *δ* (ppm): 167.87, 161.99 (d, J = 243.0 Hz), 158.94, 158.71, 153.45, 148.38, 142.22, 133.51 (d, J = 3.0 Hz), 131.52 (d, J = 9.0 Hz), 115.82 (d, J = 21.0 Hz), 108.42, 106.34, 101.63, 101.63, 98.60, 67.36, 37.36; Anal. calcd. for m/z of C_18_H_14_FN_3_O_4_S_2_ (HRMS [M + Na]^+^): 442.03020, found: 442.02960.

2-(Benzo[d][1,3]dioxol-5-yloxy)-N-(5-((4-chlorobenzyl)thio)-1,3,4-thiadiazol-2-yl)acetamide (**5s**). Yellow solid, yield 70%, mp 166–168°C; ^1^H NMR (400 MHz, DMSO-d_6_) *δ* (ppm): 12.84 (s, 1H), 7.45–7.38 (m, 4H), 6.82 (d, J = 8.0 Hz, 1H), 6.71 (d, J = 4.0 Hz, 1H), 6.40 (q, J1 = 4.0 Hz, J2 = 8.0 Hz, 1H), 5.97 (s, 2H), 4.82 (s, 2H), 4.49 (s, 2H); ^13^C NMR (100 MHz, DMSO-d_6_) *δ* (ppm): 167.88, 158.99, 158.58, 153.45, 148.38, 142.22, 136.46; Anal. calcd. for m/z of C_18_H_14_ClN_3_O_4_S_2_ (HRMS [M + Na]^+^): 458.00065, found: 458.00023.

### 3.2 Biological evaluations

Table 1 showed that, at 100 and 50 μg/ml, compounds 5a, 5b, 5d, 5e, 5f, 5g, 5k, 5L, and 5n showed significant *in vitro* inhibitory effect against Xac, with the inhibition rate ranges of 46%–84% and 33%–70%, respectively, which were higher than thiodiazole copper and bismerthiazol. Meanwhile, compounds 5a, 5b, 5f, 5k, 5L, and 5n exhibited excellent *in vitro* antibacterial activity against Xoc, with the inhibition rate ranges of 71%–92% and 53%–80% at 100 and 50 μg/ml, respectively, which were superior to thiodiazole copper and bismerthiazol. In particular, Table 2 showed that the EC_50_ values for compound 2-(2-methoxyphenoxy)-N-(5-(methylthio)-1,3,4-thiadiazol-2-yl)acetamide (5k) against Xac and Xoc were 22 and 15 μg/ml, respectively, which were higher than thiodiazole copper and bismerthiazol.

**TABLE 1:** *In vitro* antibacterial activity test of compounds 5a−5s against Xac and Xoc.

Compounds	Inhibition rate (%)^a^			
	Xac		Xoc	
	100 μg/ml	50 μg/ml	100 μg/ml	50 μg/ml
5a	76 ± 2.21	60 ± 1.14	82 ± 1.29	67 ± 1.85
5b	67 ± 1.14	51 ± 1.74	74 ± 2.01	61 ± 2.04
5c	35 ± 2.00	28 ± 1.11	42 ± 0.94	30 ± 1.24
5d	54 ± 2.11	42 ± 1.01	62 ± 1.59	40 ± 2.14
5e	46 ± 2.95	33 ± 1.01	54 ± 1.94	31 ± 1.54
5f	62 ± 1.19	50 ± 1.10	73 ± 2.49	55 ± 1.29
5g	51 ± 1.09	40 ± 0.59	64 ± 1.95	40 ± 0.74
5h	32 ± 1.50	21 ± 1.51	36 ± 2.49	28 ± 1.64
5i	47 ± 1.14	33 ± 2.04	55 ± 2.17	36 ± 1.74
5j	37 ± 0.49	25 ± 2.06	48 ± 2.10	32 ± 1.75
5k	84 ± 1.06	70 ± 1.96	92 ± 1.49	80 ± 1.91
5L	75 ± 1.11	61 ± 1.33	84 ± 1.86	70 ± 1.65
5m	30 ± 1.22	18 ± 2.01	52 ± 1.57	41 ± 2.56
5n	52 ± 1.74	40 ± 2.08	71 ± 1.74	53 ± 2.49
5o	35 ± 1.27	22 ± 1.84	60 ± 1.04	45 ± 1.99
5p	10 ± 2.04	4 ± 1.19	16 ± 2.22	8 ± 1.64
5q	0	0	0	0
5r	0	0	8 ± 2.14	2 ± 0.54
5s	0	0	0	0
Bismerthiazol^b^	38 ± 2.02	30 ± 2.04	67 ± 1.54	42 ± 2.01
Thiodiazole copper^b^	30 ± 2.01	20 ± 1.62	52 ± 1.94	37 ± 1.86

a Average of three times for each treatment.

b The positive control.

**TABLE 2:** The EC_50_ values of compounds 5a, 5b, 5f, 5k, 5L, and 5n against Xac and Xoc.

Compounds	Inhibition rate (%)^a^	
	Xac	Xoc
5a	30 ± 1.25	23 ± 2.21
5b	40 ± 2.65	32 ± 1.65
5f	45 ± 1.94	35 ± 1.26
5k	22 ± 1.54	15 ± 1.62
5L	28 ± 1.24	20 ± 0.98
5n	50 ± 2.28	41 ± 1.97
Bismerthiazol^b^	142 ± 2.26	65 ± 3.24
Thiodiazole copper^b^	181 ± 4.65	102 ± 2.18

a Average of three times for each treatment.

b The positive control.

Table 3 showed that compounds 5a−5s revealed lower *in vitro* inhibitory effects against M. bainieri, M. fragilis, and T. atroviride, with the inhibition rate ranges of 0%–51%, 0%–47%, and 0%–21% at 50 μg/ml, respectively, than carbendazim.

**TABLE 3:** *In vitro* antifungal activity test of compounds **5a**−**5s** against M. bainieri, M. fragilis, and T. atroviride.

Compounds	Inhibition rate (%)^a^		
	M. bainieri	M. fragilis	T. atroviride
5a	42 ± 1.54	36 ± 1.26	11 ± 0.25
5b	21 ± 1.25	14 ± 2.49	0
5c	0	0	0
5d	10 ± 1.02	0	0
5e	0	0	0
5f	20 ± 1.42	15 ± 1.26	9 ± 2.24
5g	12 ± 2.01	10 ± 2.28	0
5h	0	0	0
5i	2 ± 1.01	0	0
5j	0	0	0
5k	51 ± 1.04	47 ± 1.65	21 ± 1.36
5L	30 ± 1.11	25 ± 2.21	9 ± 2.46
5m	8 ± 1.64	0	0
5n	20 ± 0.84	12 ± 0.54	0
5o	13 ± 2.17	0	0
5p	16 ± 1.28	12 ± 1.05	2 ± 1.10
5q	0	0	0
5r	0	0	0
5s	0	0	0
Carbendazim^b^	100	100	100

a Average of three times for each treatment.

b The positive control.

### 3.3 Structure-activity relationship analysis

The SAR analysis was analyzed based on the inhibitory activity listed in Tables 1 and 2. First, the presence of the 2-OCH_3_ group at R_1_ substituent group showed better inhibitory activity in the order of 5k > 5f, 5k > 5a, and 5k > 5p. Second, the CH_3_ group at the R_2_ substituent group could increase the inhibitory activity followed the order of 5a > 5b, 5f > 5g, and 5k > 5L.

## 4 Conclusion

In conclusion, using the botanical active components of carvacrol, thymol, guaiacol, and sesamol as the lead structures, 19 structurally characterized botanical active component derivatives containing carboxamide and 1,3,4-thiadiazole thioether moieties were prepared. Bioassay results demonstrated that compound 2-(2-methoxyphenoxy)-N-(5-(methylthio)-1,3,4-thiadiazol-2-yl)acetamide (**5k**) had the higher inhibitory activity against Xac and Xoc than thiodiazole copper and bismerthiazol. Meanwhile, the analysis of SAR results showed that the presence of the 2-OCH_3_ and CH_3_ groups at R_1_ and R_2_ substituent groups, respectively, could increase the inhibitory effects of the target compounds.

## Data Availability

The original contributions presented in the study are included in the article/Supplementary Material, further inquiries can be directed to the corresponding authors.
