# Supplementary material for: Novel botanical active component derivatives containing carboxamide and 1,3,4-Thiadiazole thioether moieties: Design, synthesis, and inhibitory activity
Source: Front Chem. 2022 Sep 27;10:1036909. doi: 10.3389/fchem.2022.1036909 (PMC9551022; doi:10.3389/fchem.2022.1036909)
Supplement: Supplementary file 1 [file DataSheet1.doc]

Supplementary Material


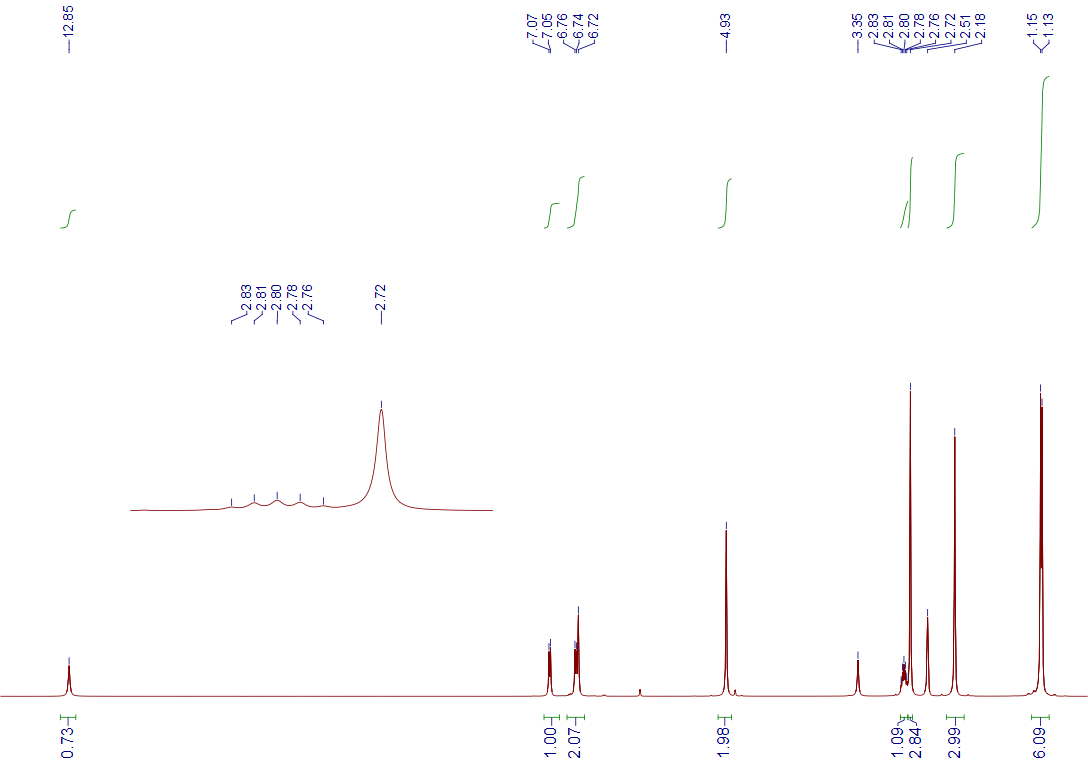


^1^H NMR of compound **5a**


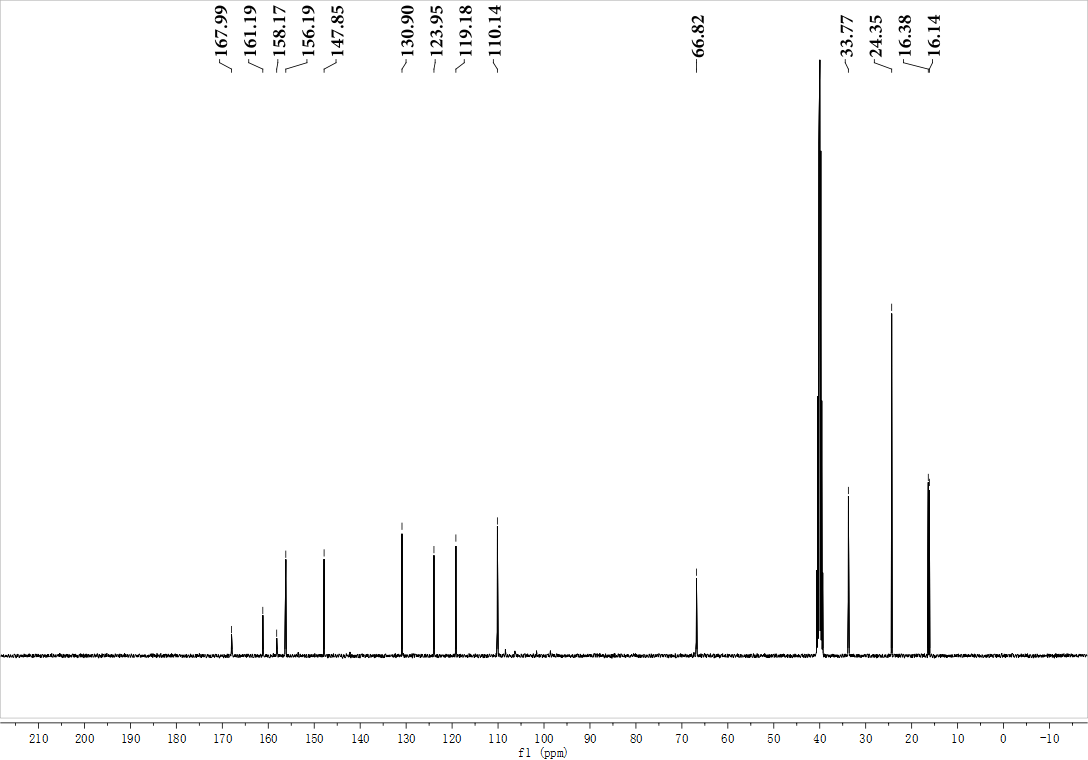


^15^C NMR of compound **5a**


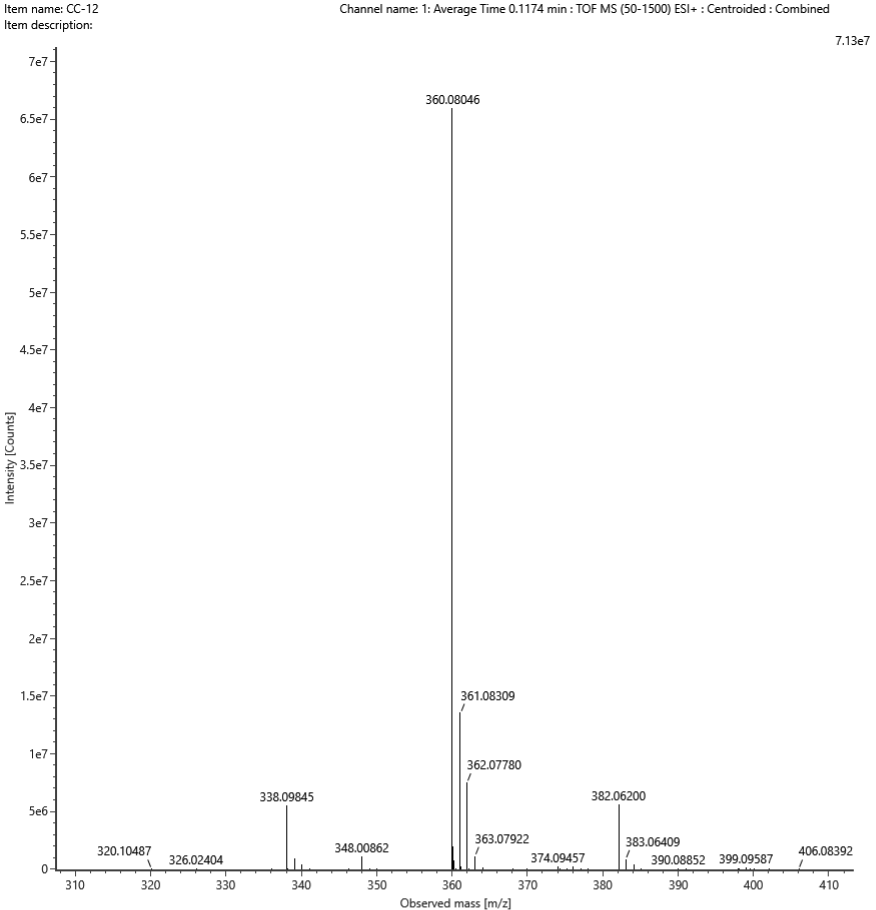


HRMS of compound **5a**


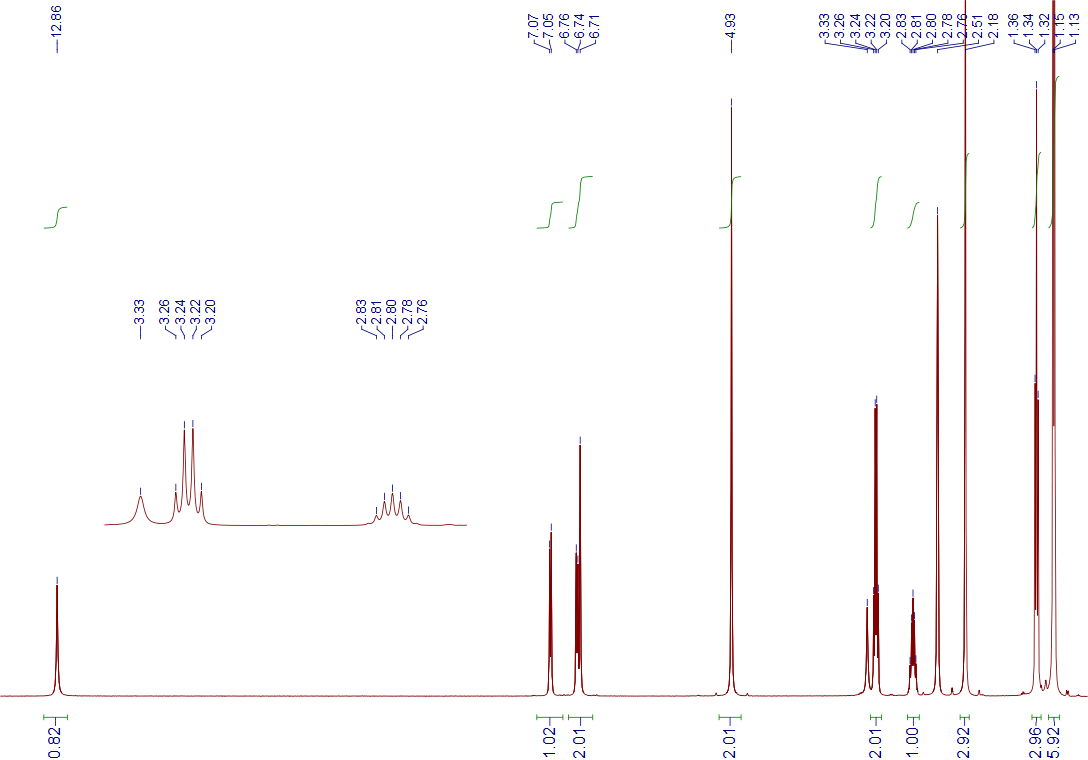


^1^H NMR of compound **5b**


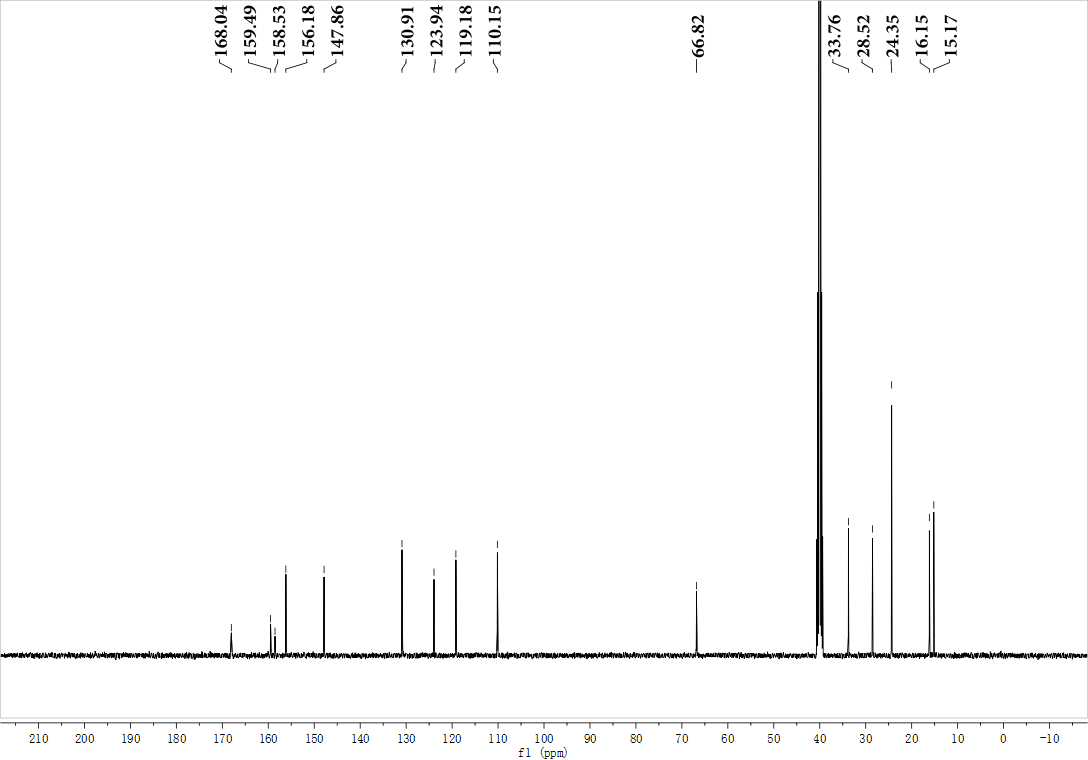


^15^C NMR of compound **5b**


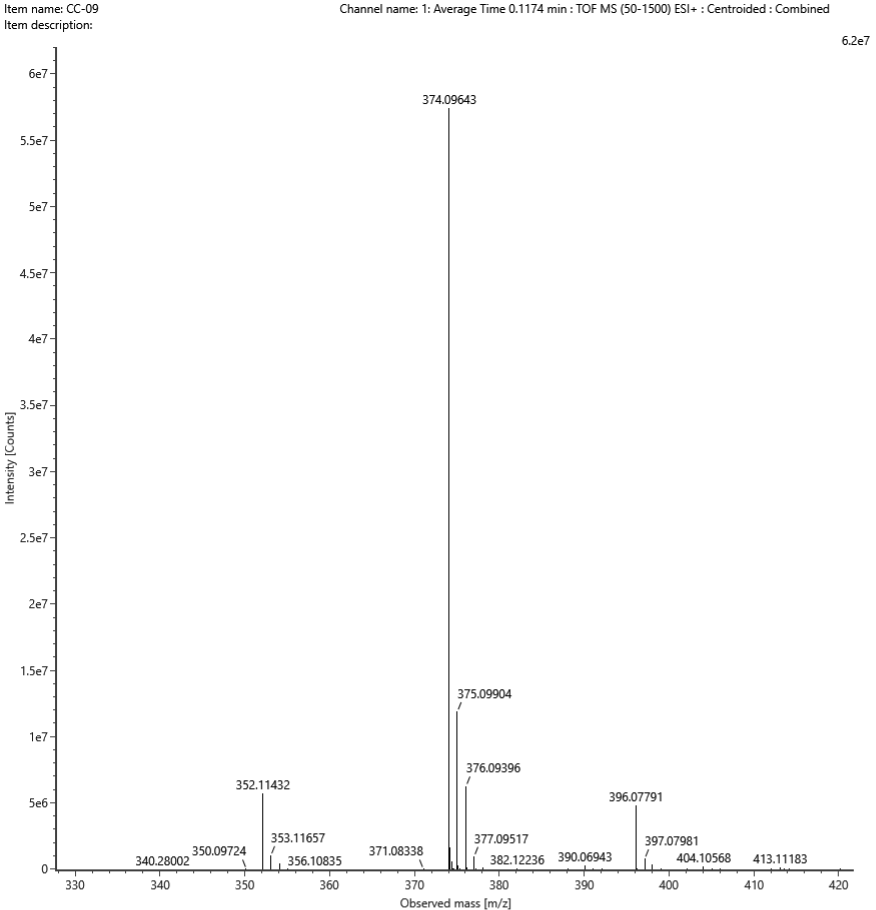


HRMS of compound **5b**


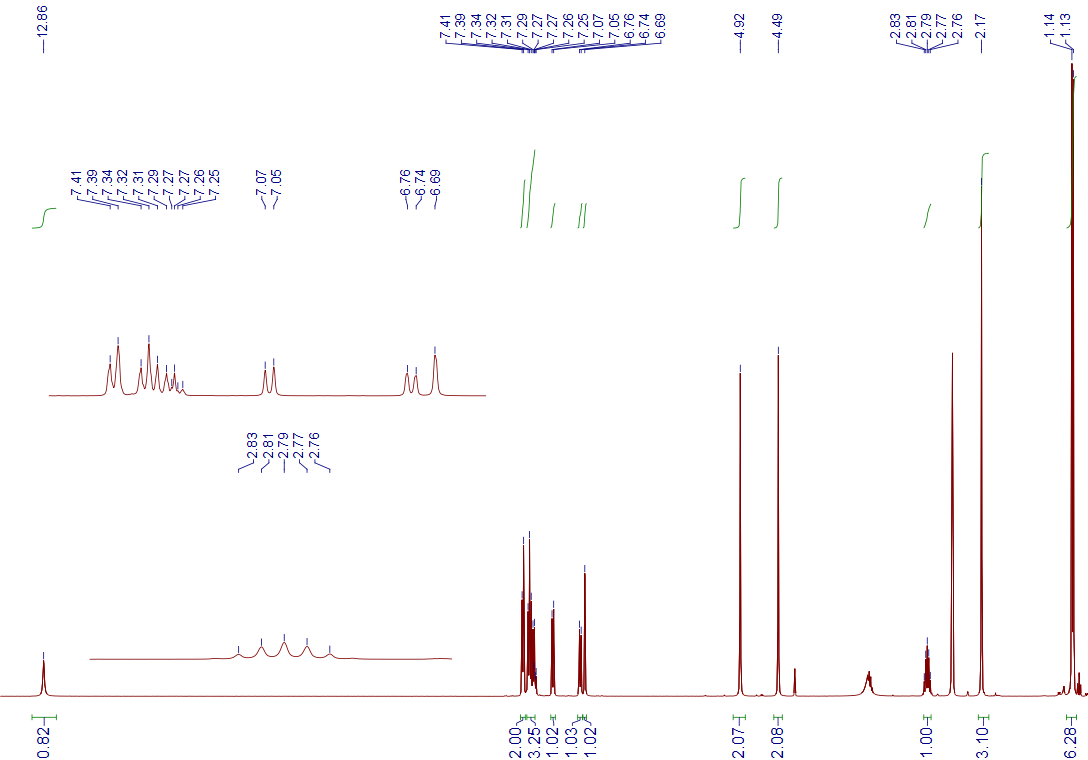


^1^H NMR of compound **5c**


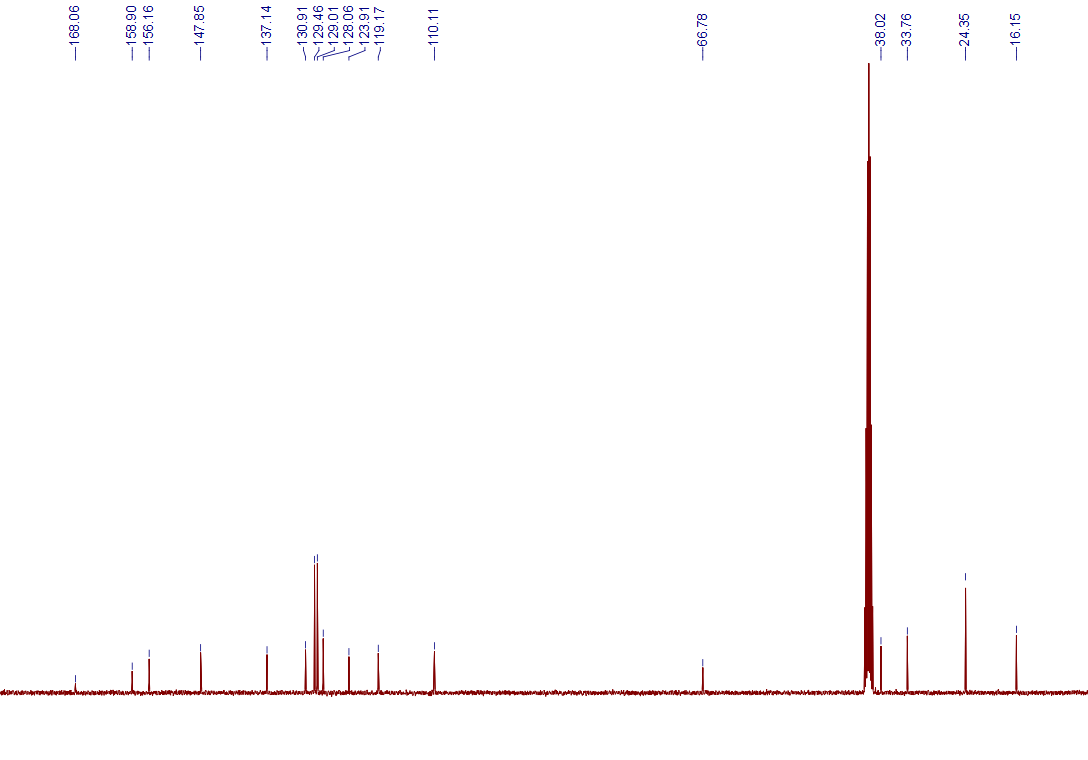


^15^C NMR of compound **5c**


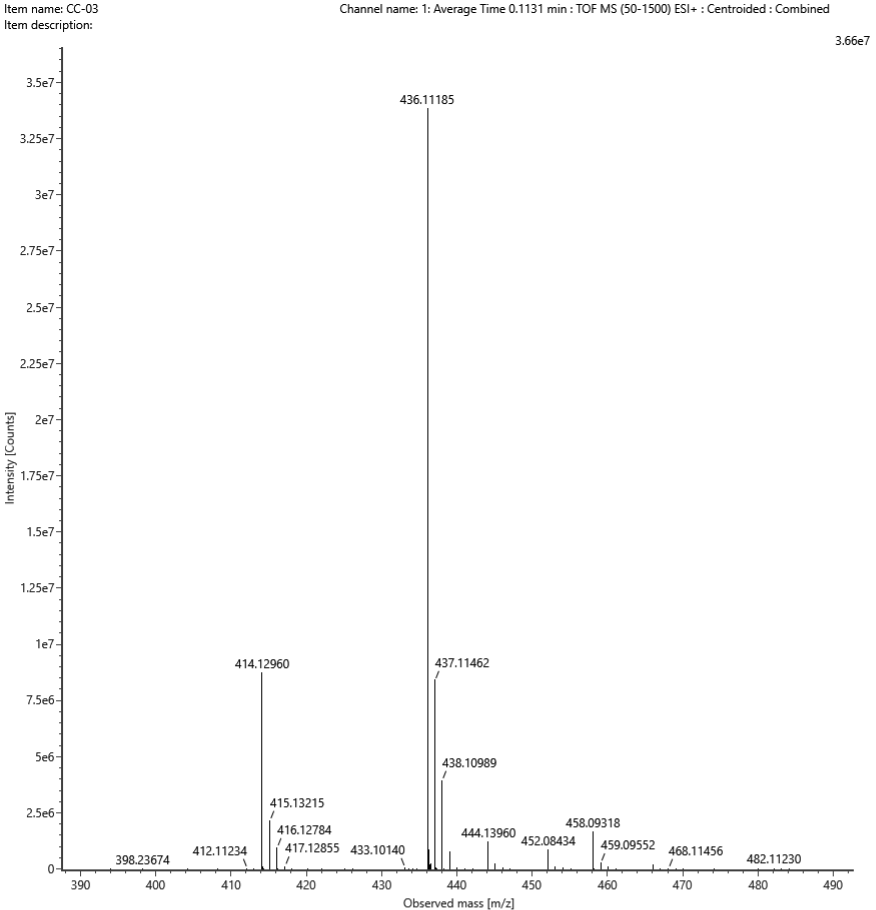


HRMS of compound **5c**


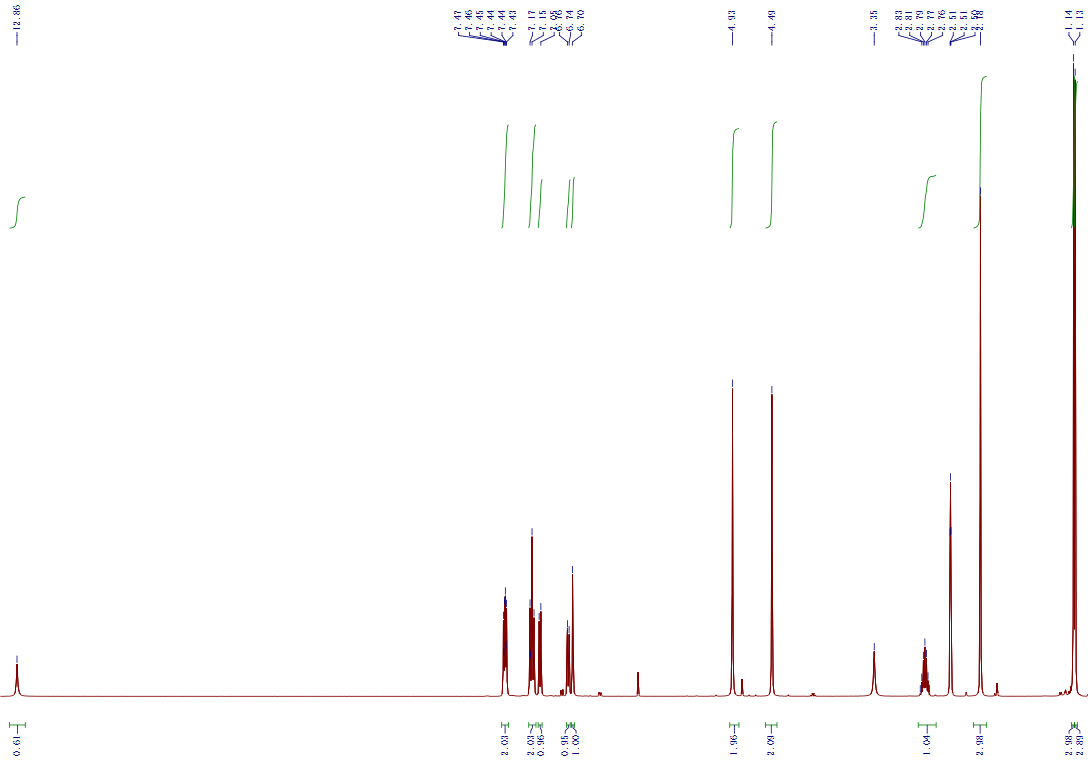


^1^H NMR of compound **5d**


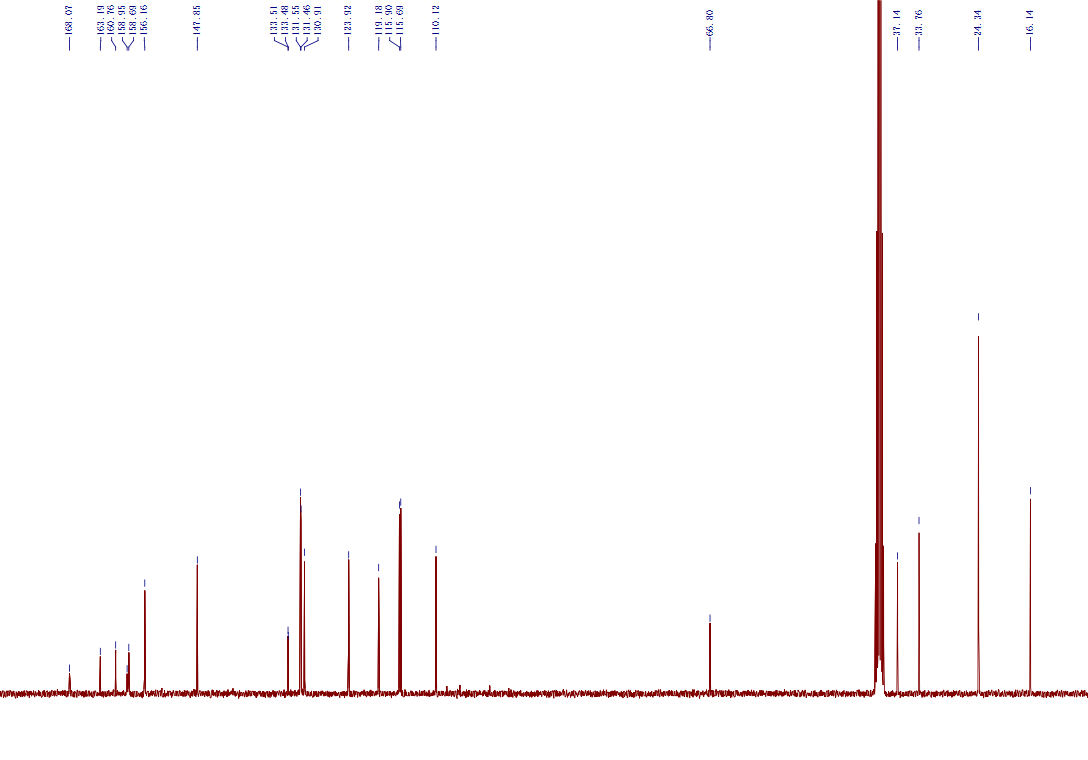


^15^C NMR of compound **5d**


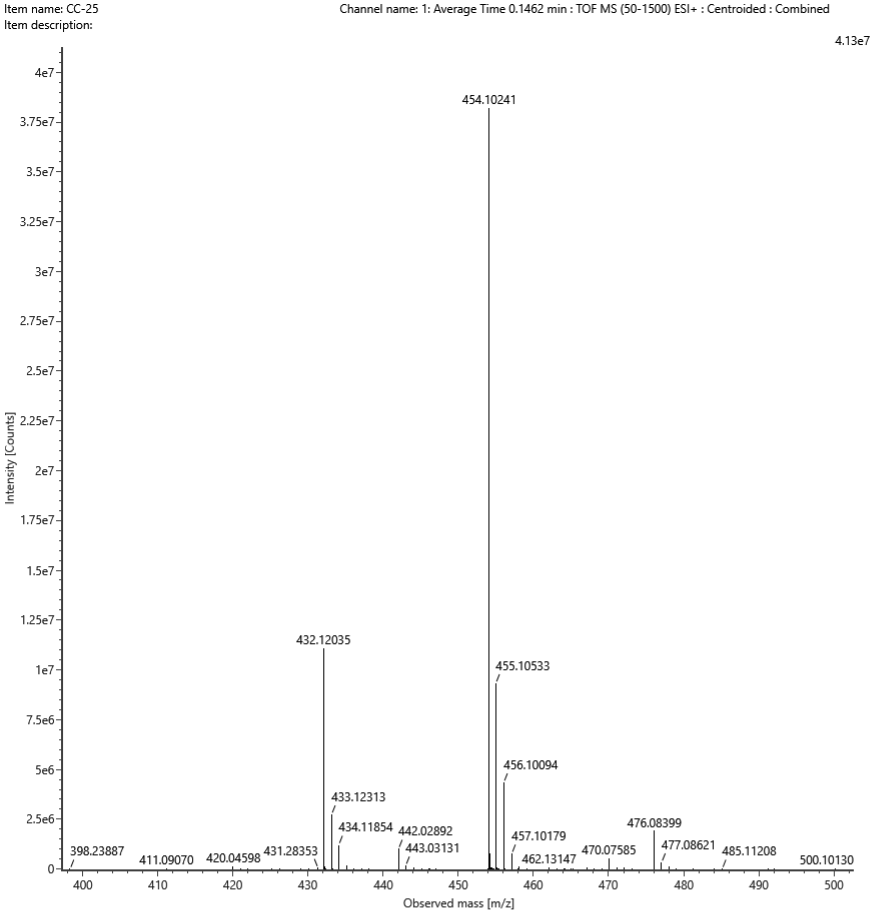


HRMS of compound **5d**


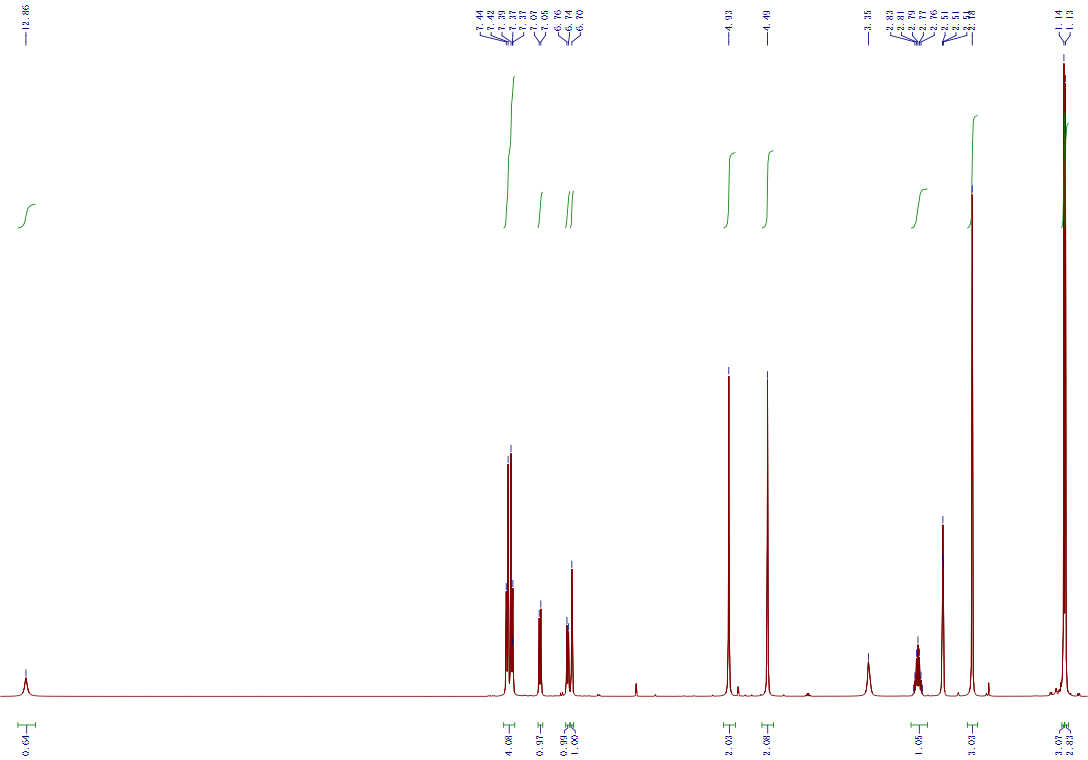


^1^H NMR of compound **5e**


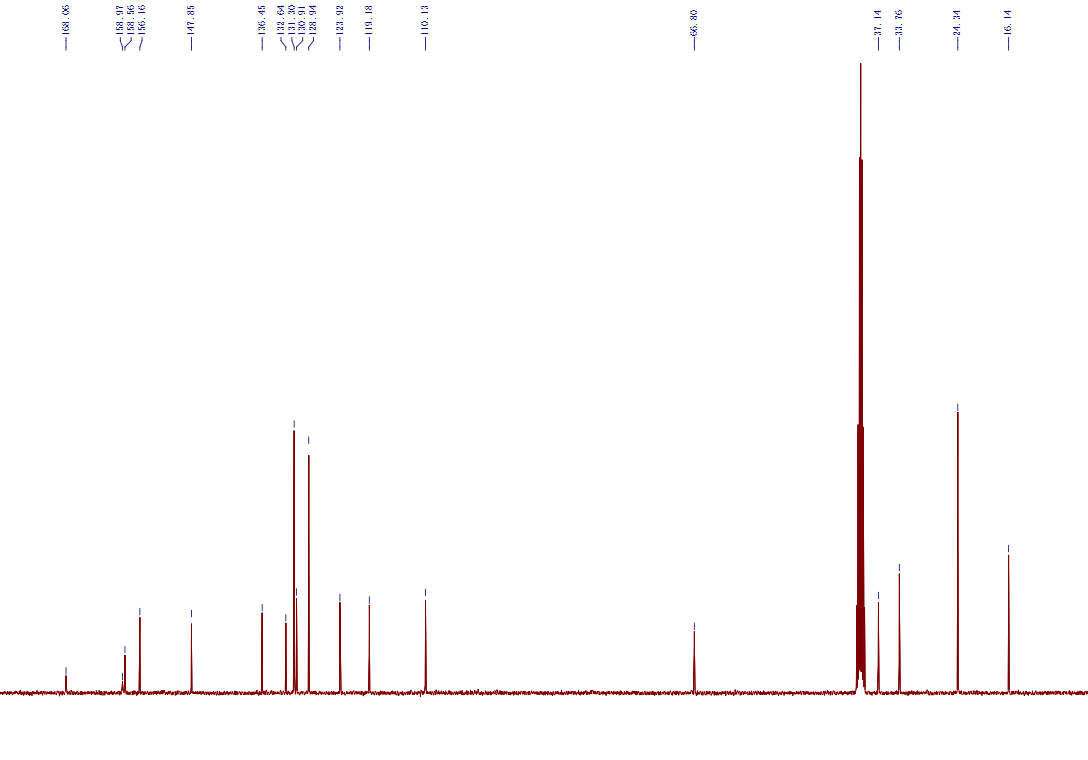


^15^C NMR of compound **5e**


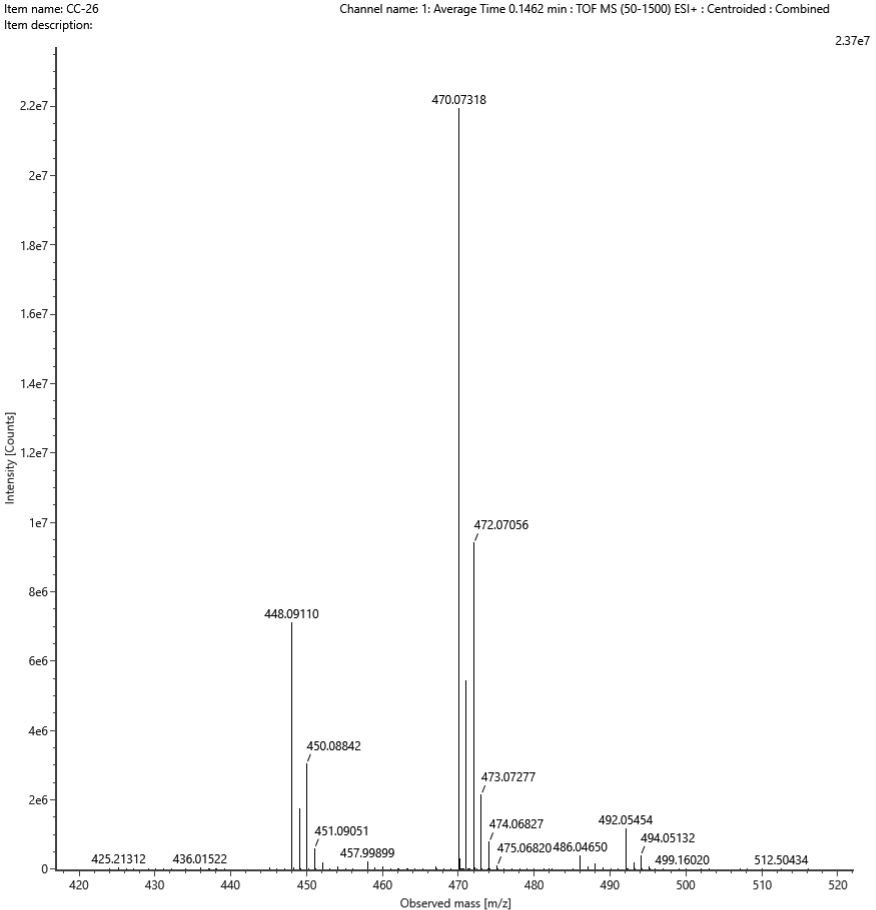


HRMS of compound **5e**


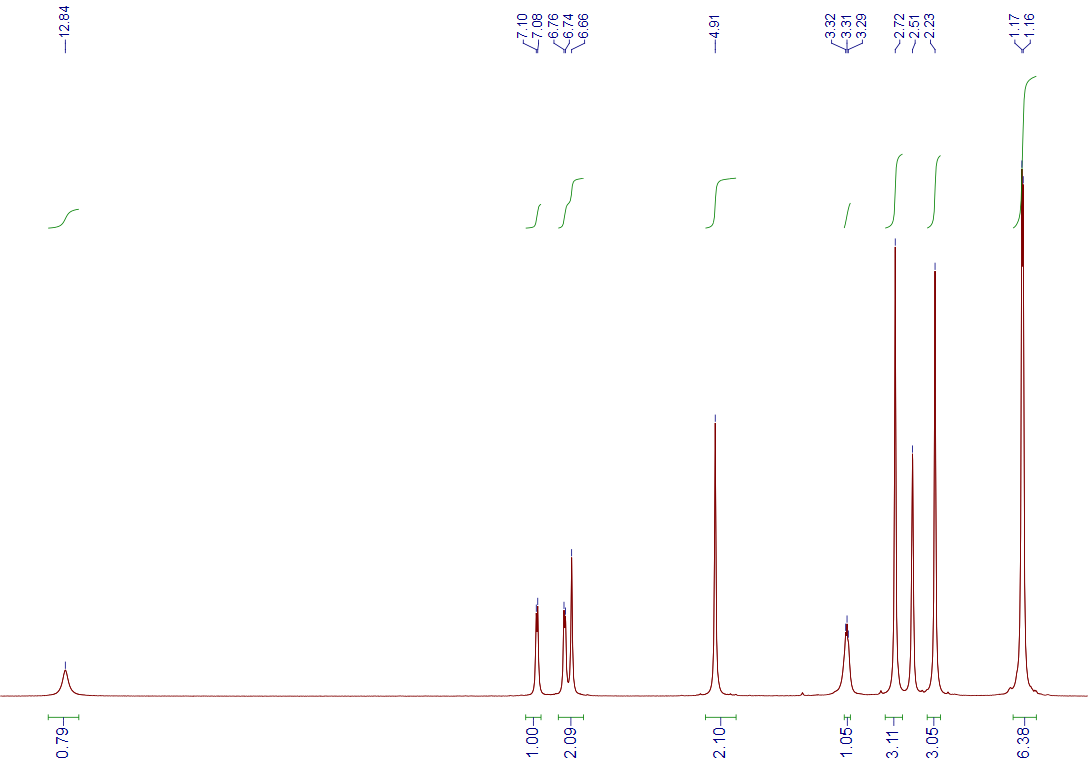


^1^H NMR of compound **5f**


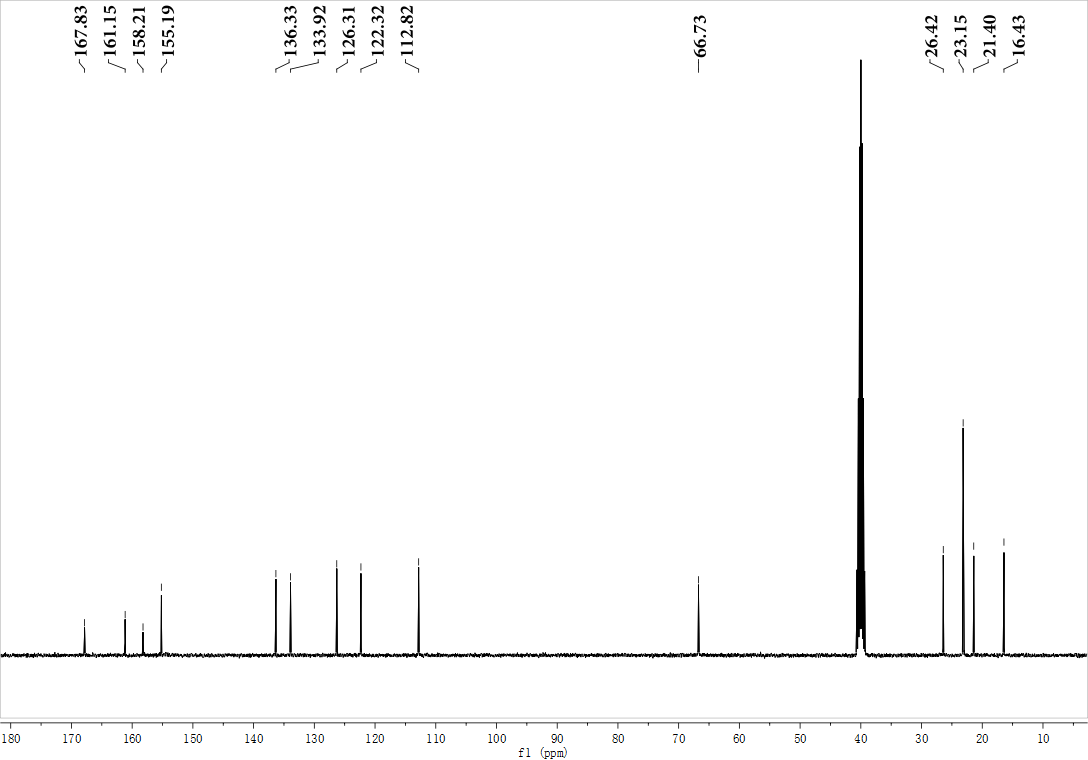


^15^C NMR of compound **5f**


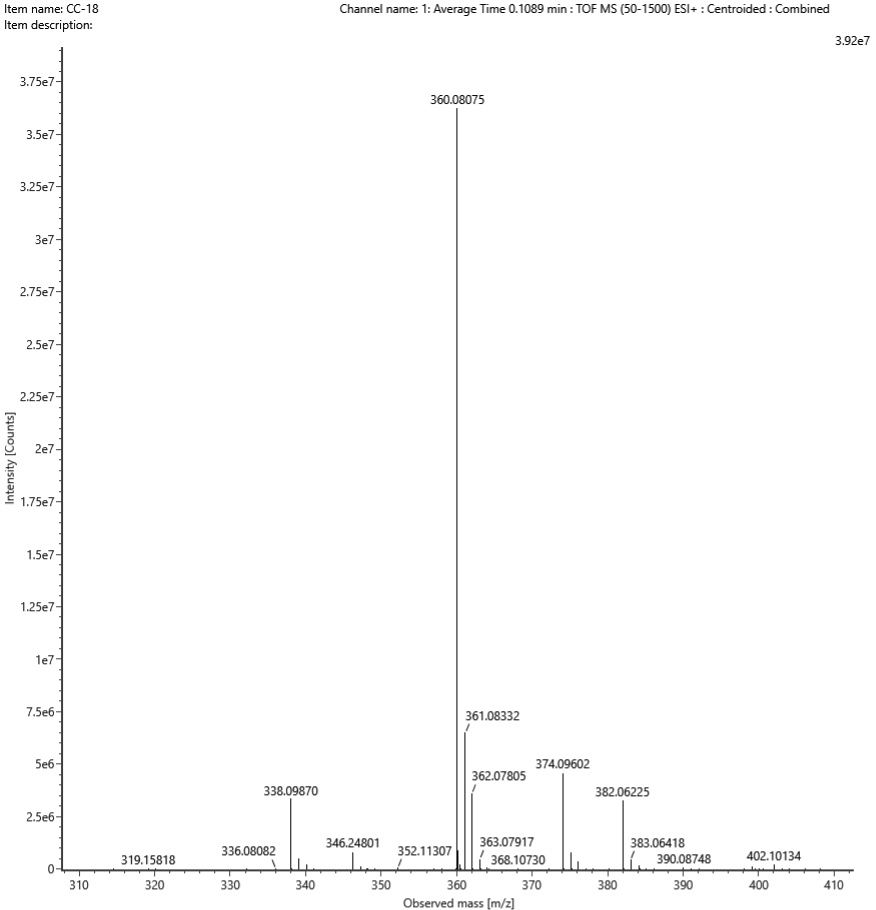


HRMS of compound **5f**


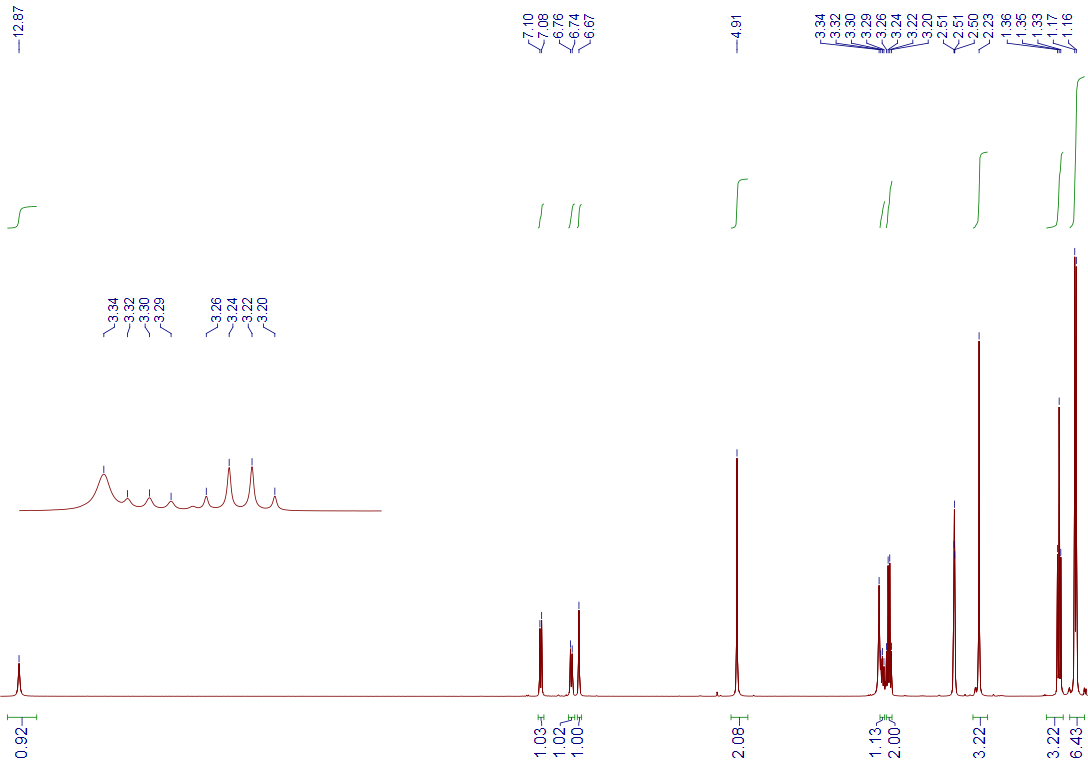


^1^H NMR of compound **5g**


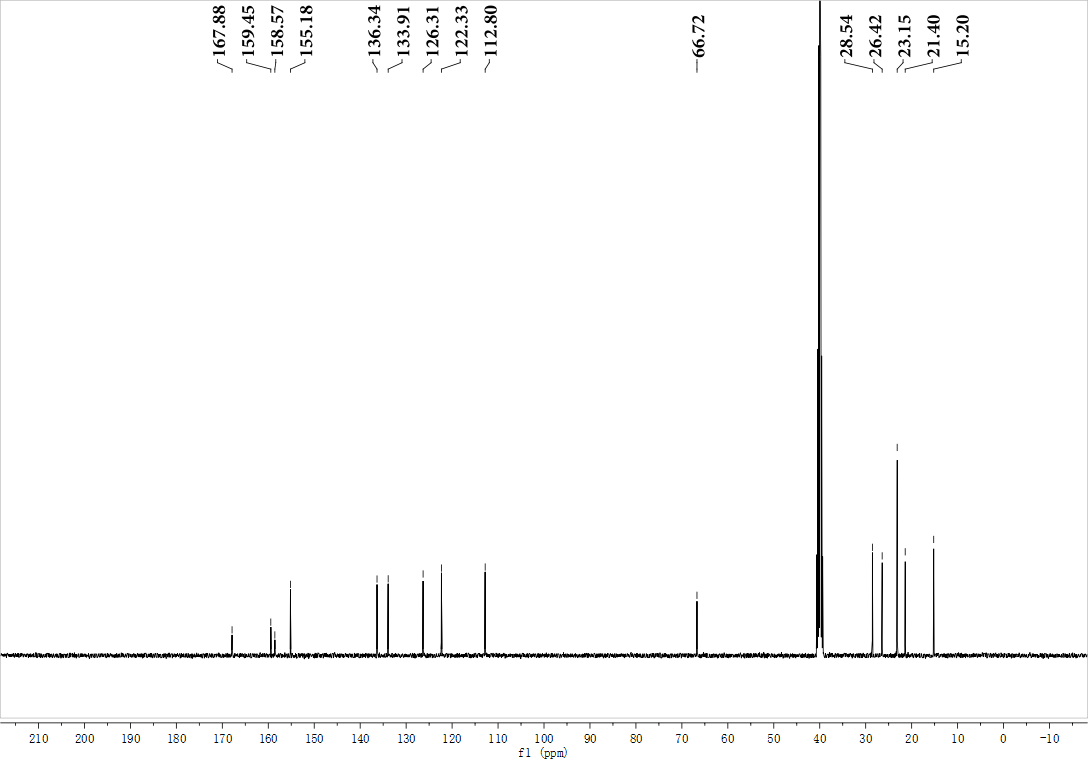


^15^C NMR of compound **5g**


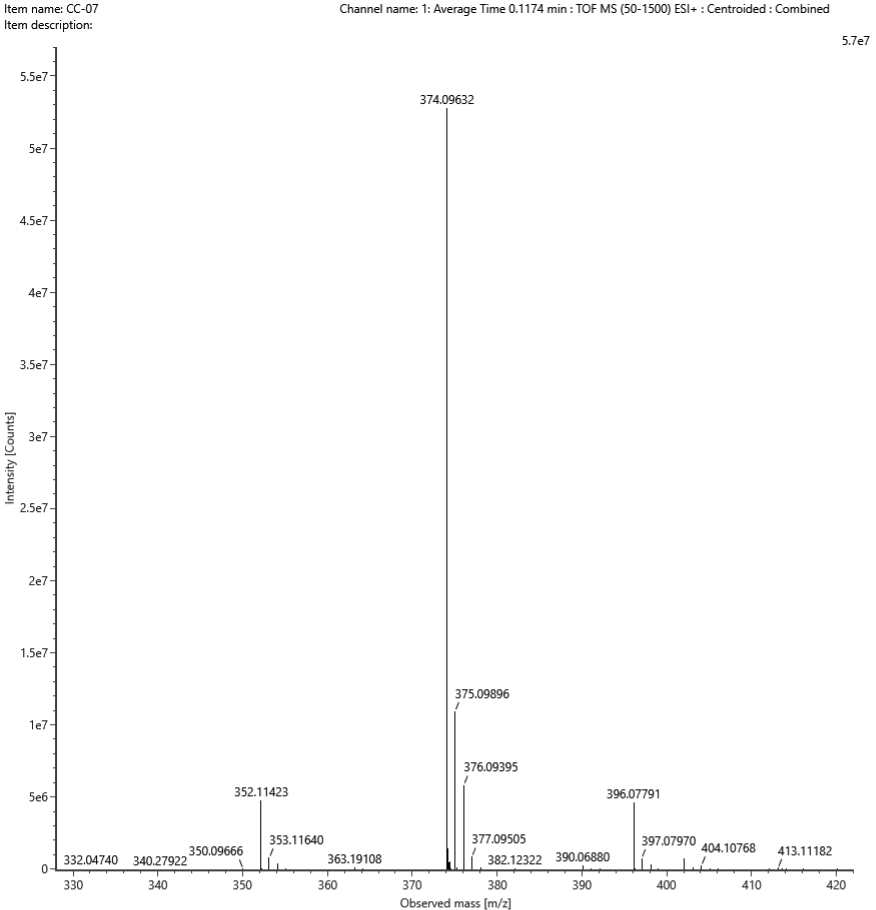


HRMS of compound **5g**


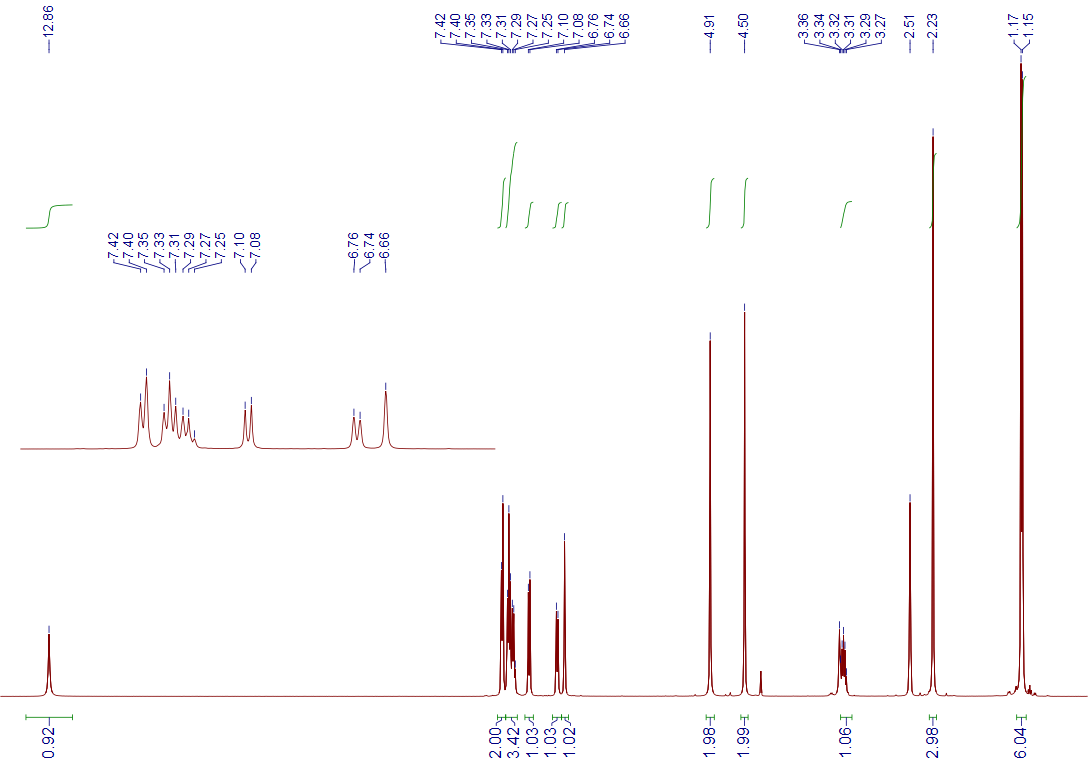


^1^H NMR of compound **5h**


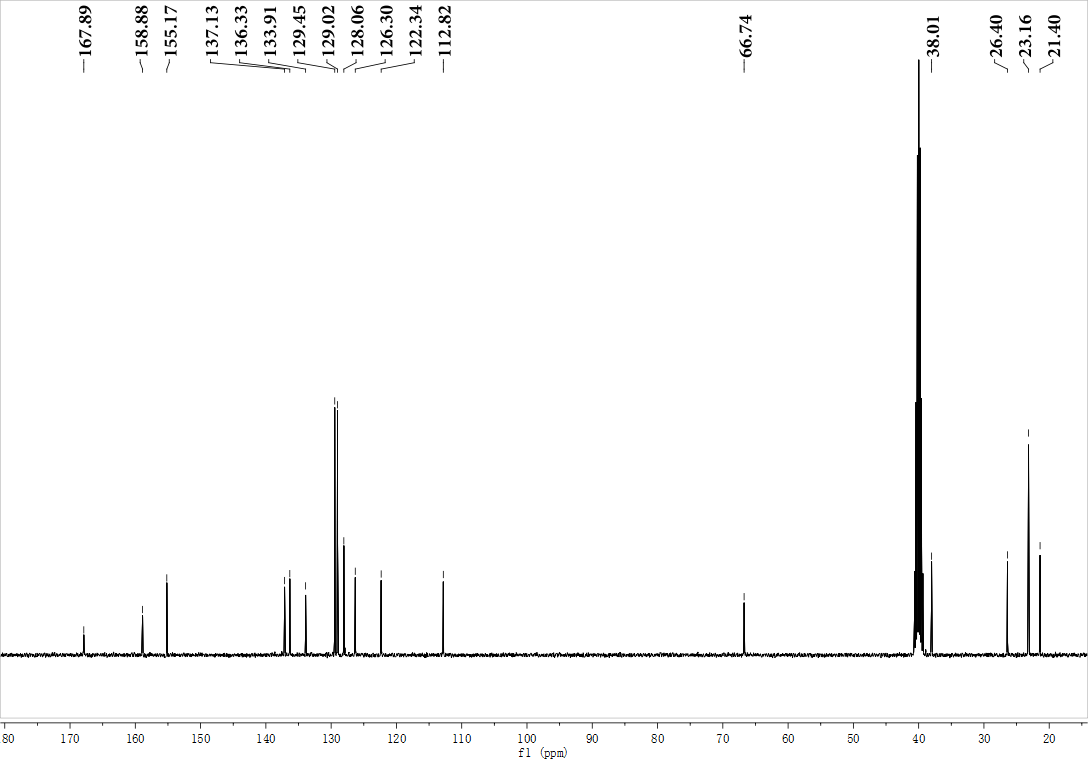


^15^C NMR of compound **5h**


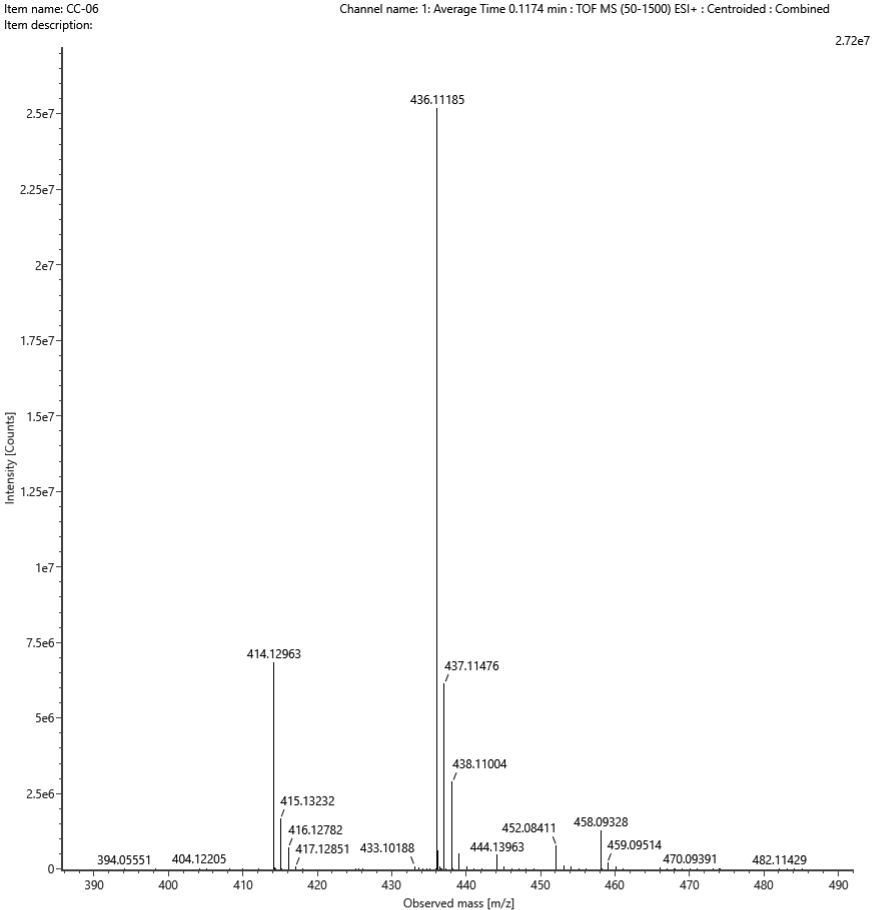


HRMS of compound **5h**


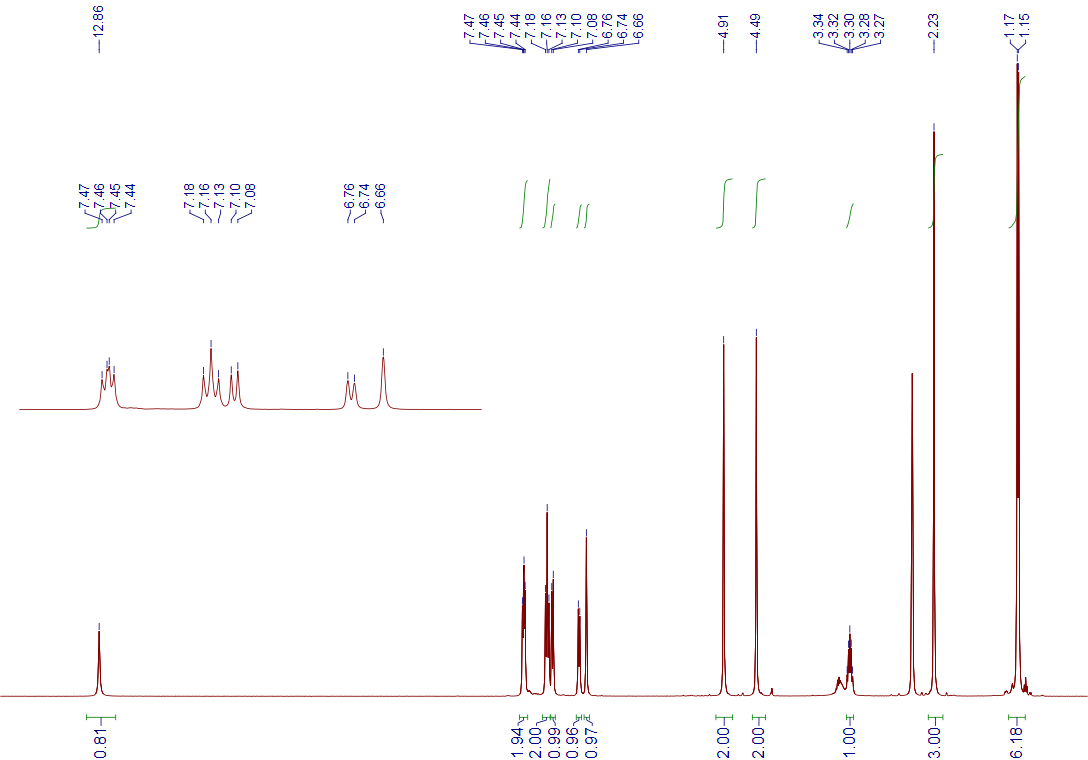


^1^H NMR of compound **5i**


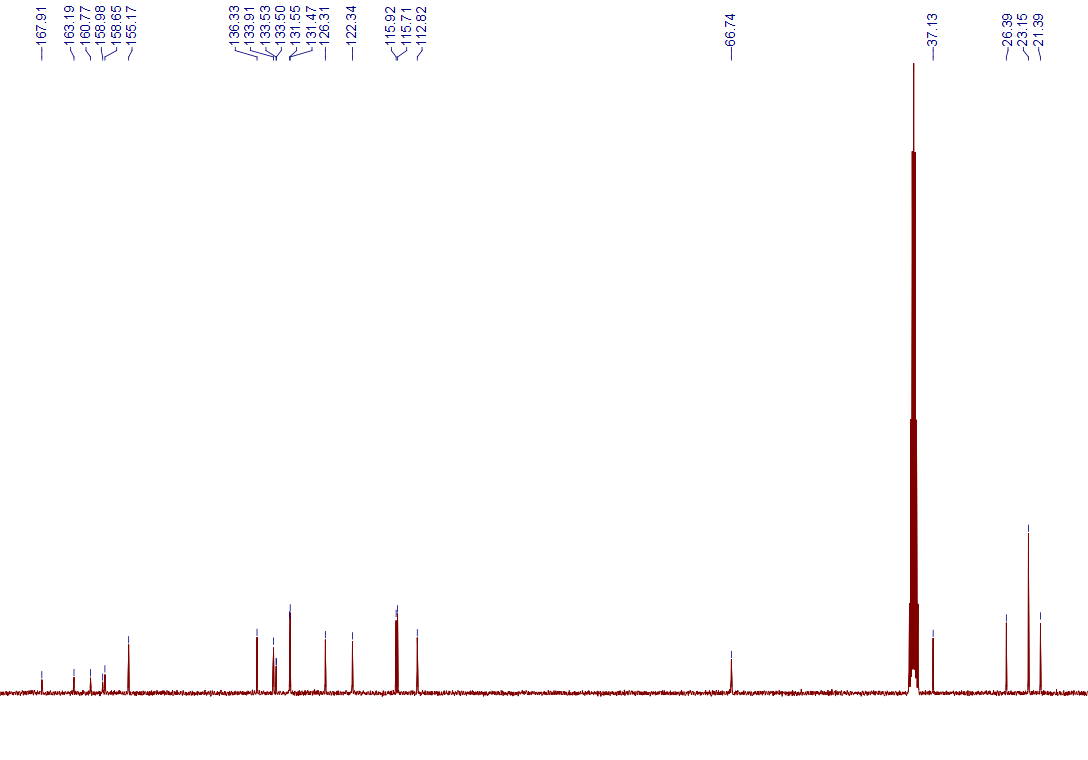


^15^C NMR of compound **5i**


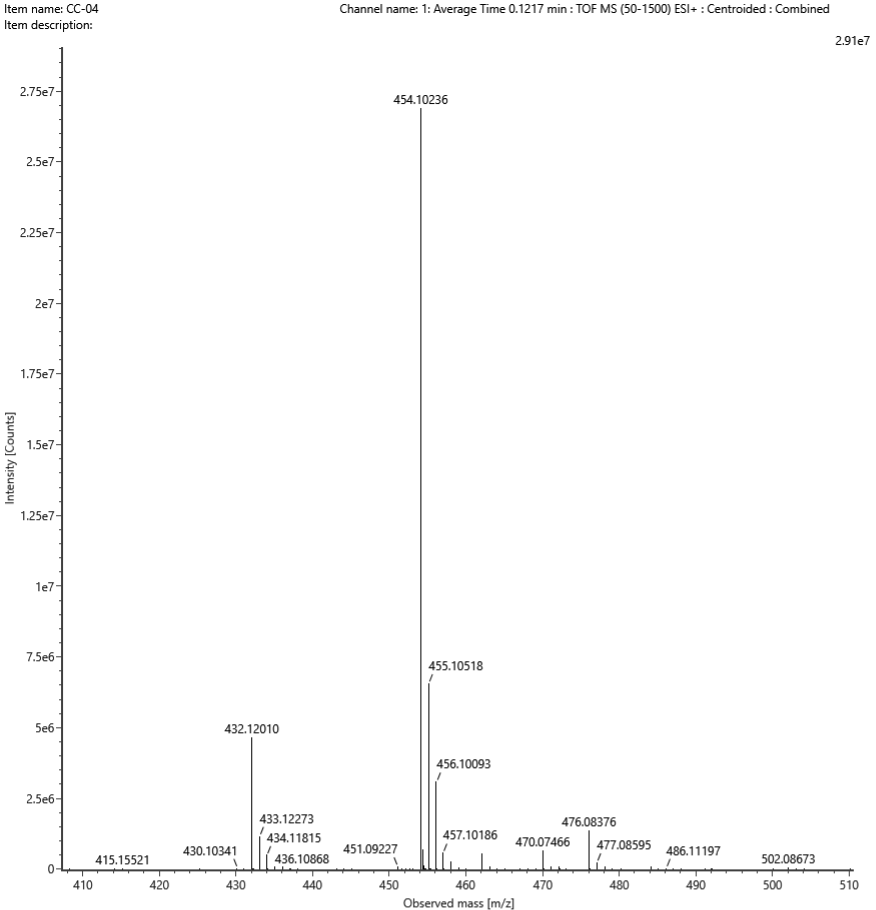


HRMS of compound **5i**


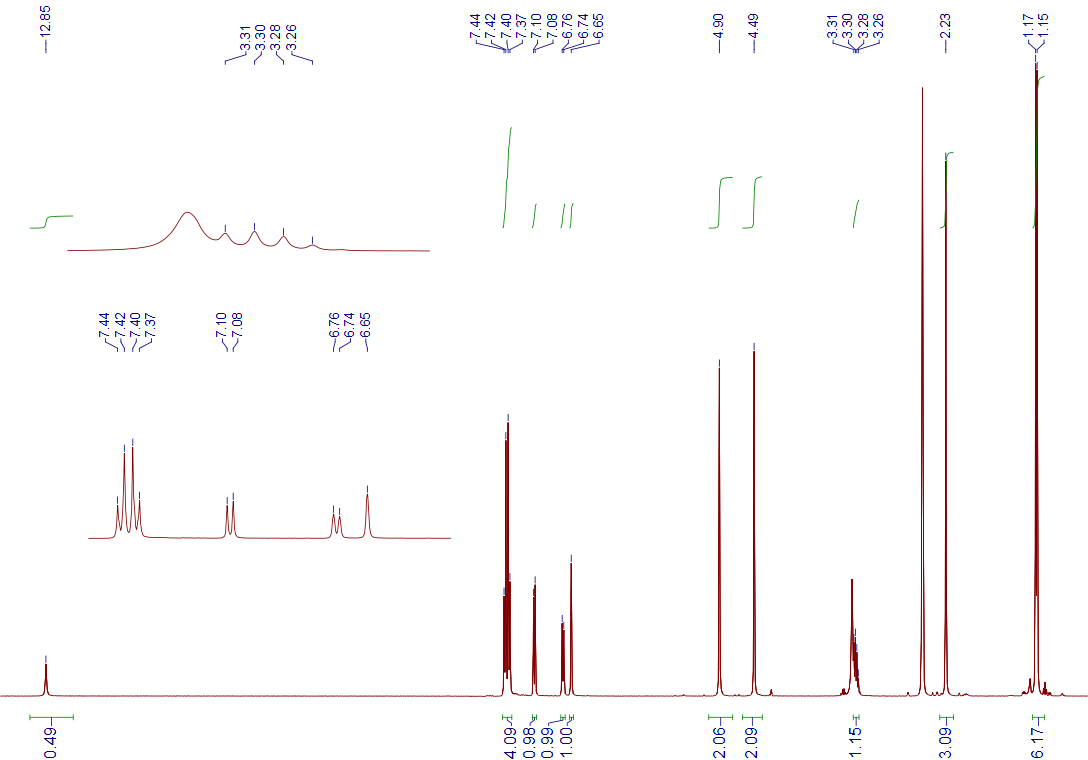


^1^H NMR of compound **5j**


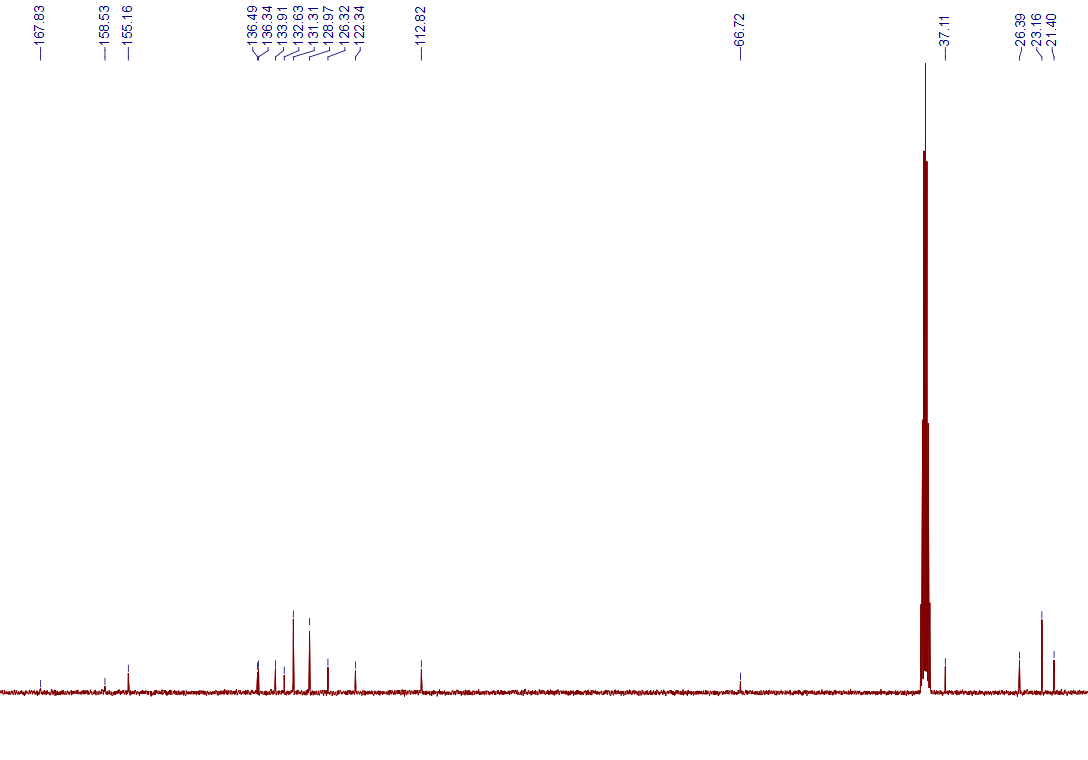


^15^C NMR of compound **5j**


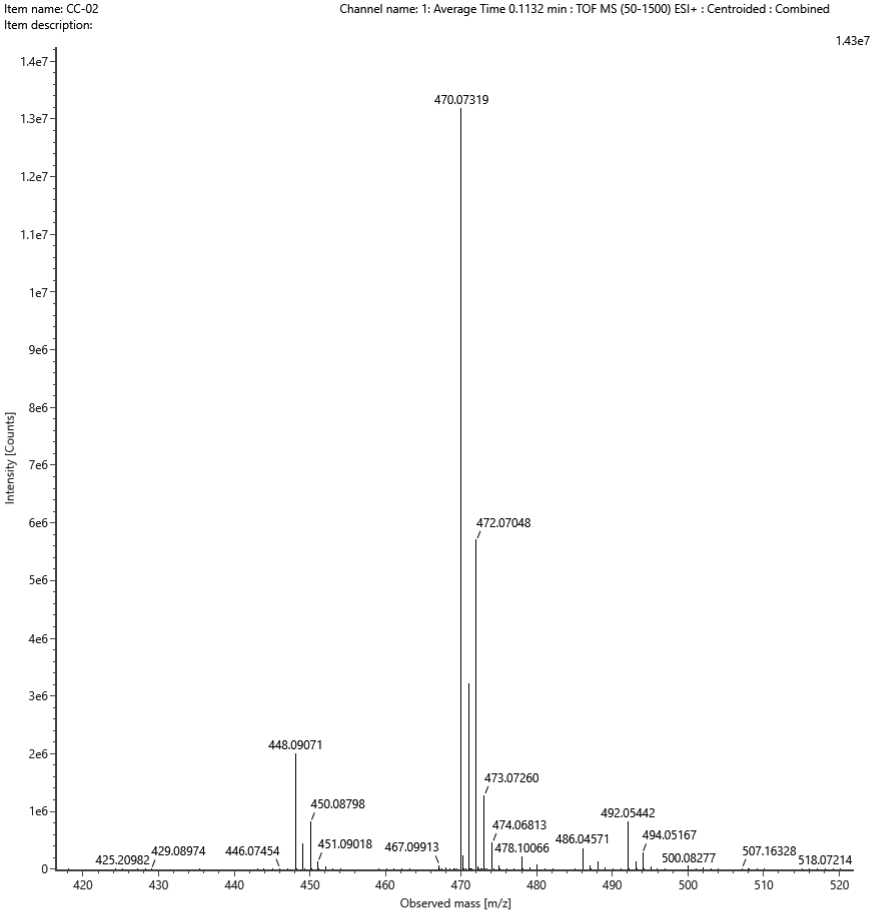


HRMS of compound **5j**


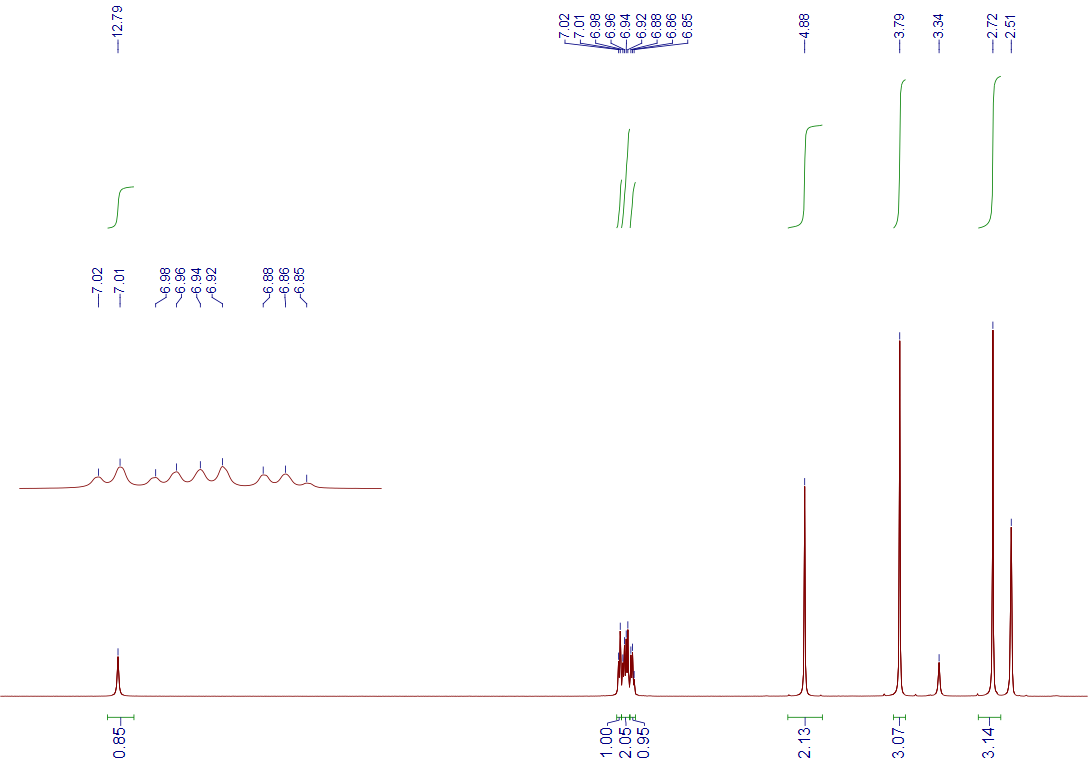


^1^H NMR of compound **5k**


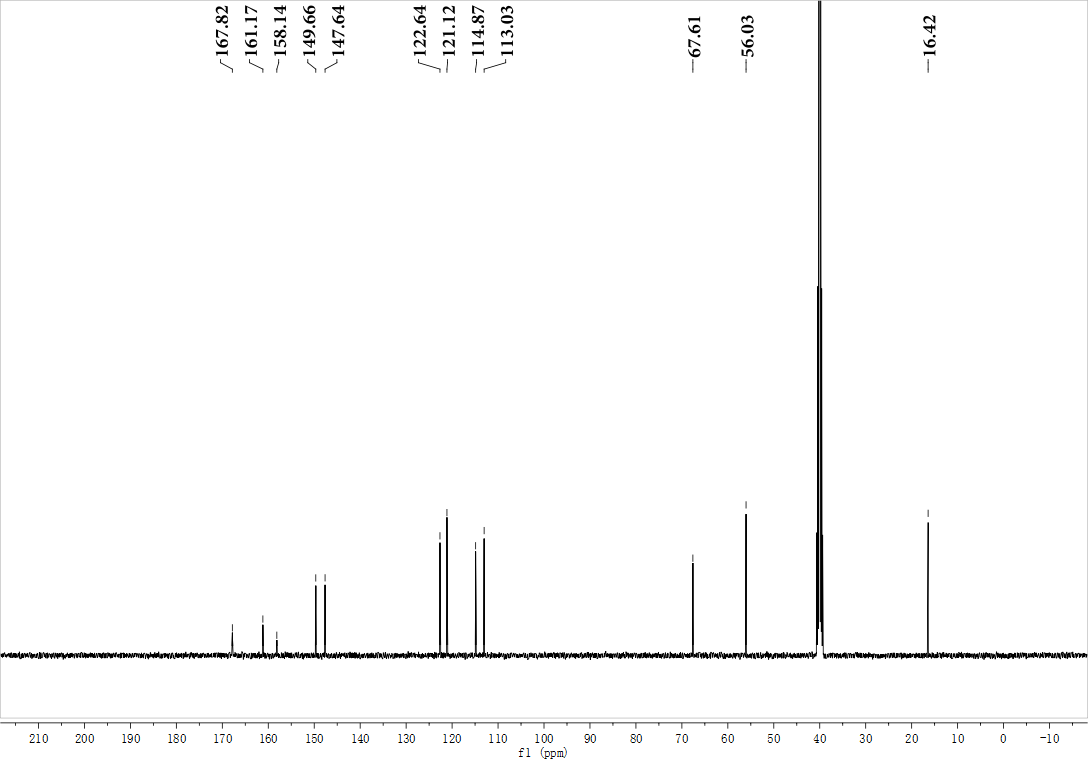


^15^C NMR of compound **5k**


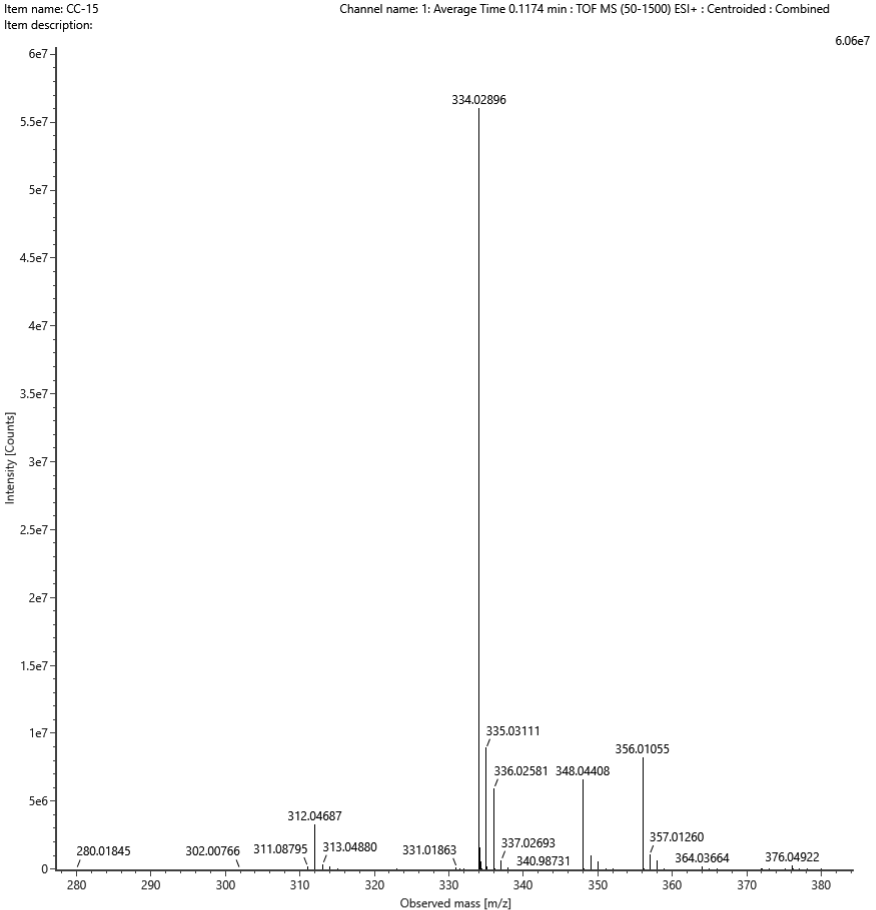


HRMS of compound **5k**


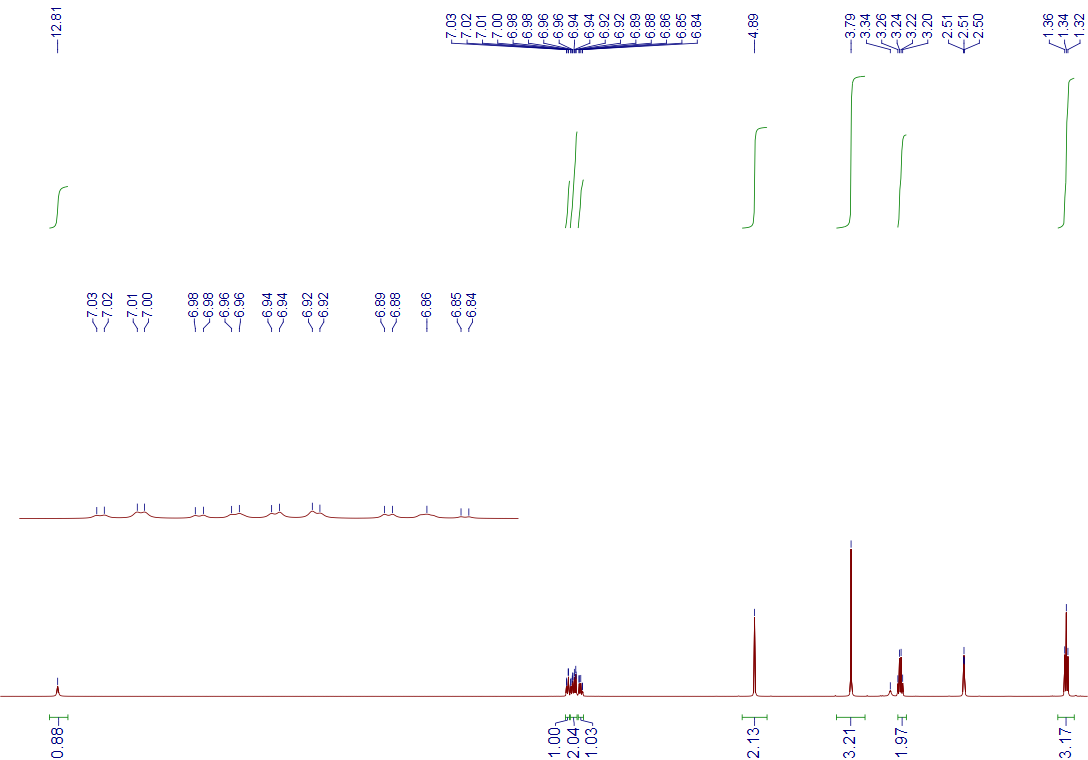


^1^H NMR of compound **5l**


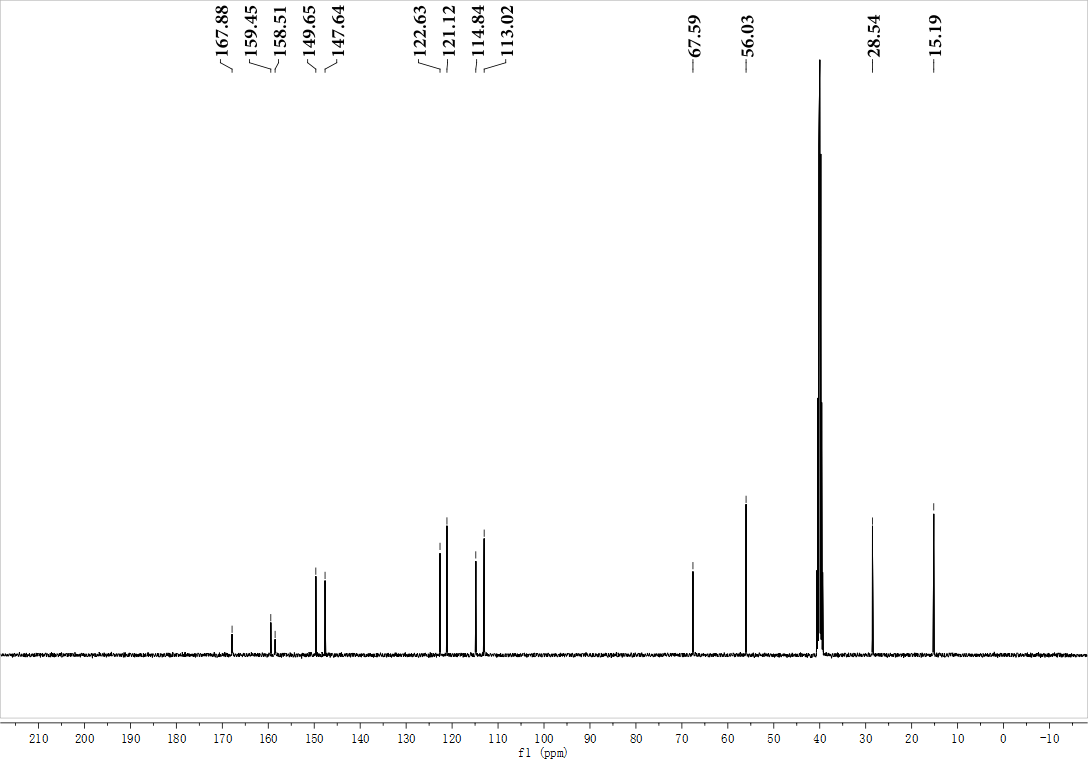


^15^C NMR of compound **5l**


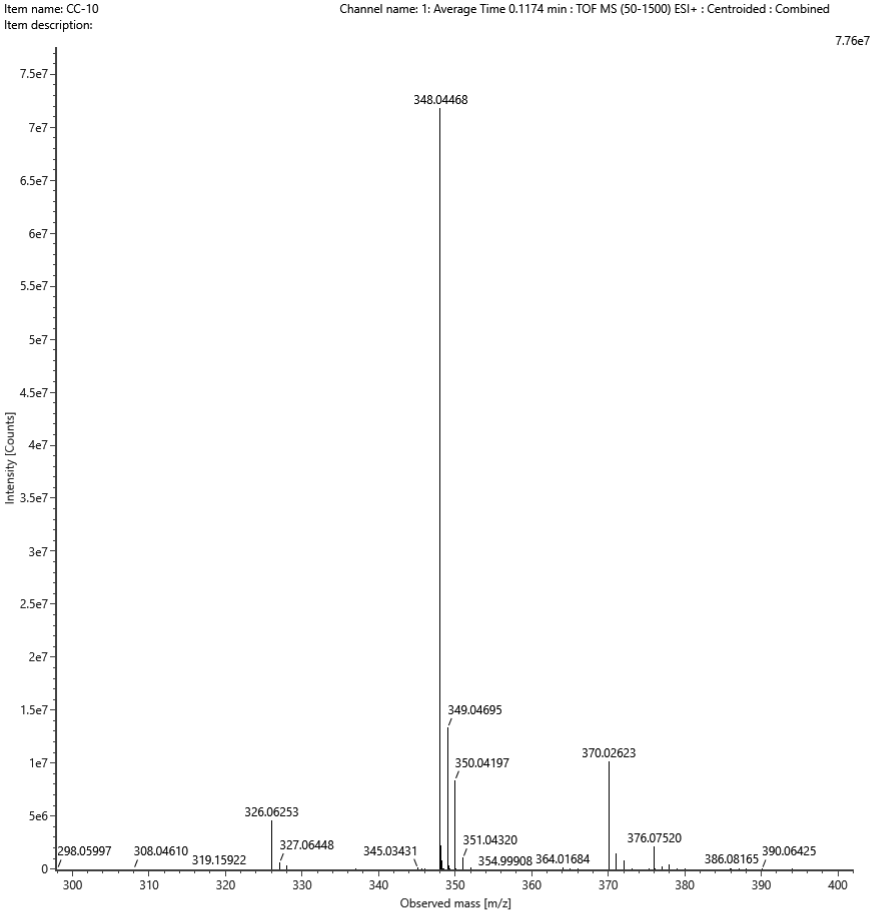


HRMS of compound **5l**


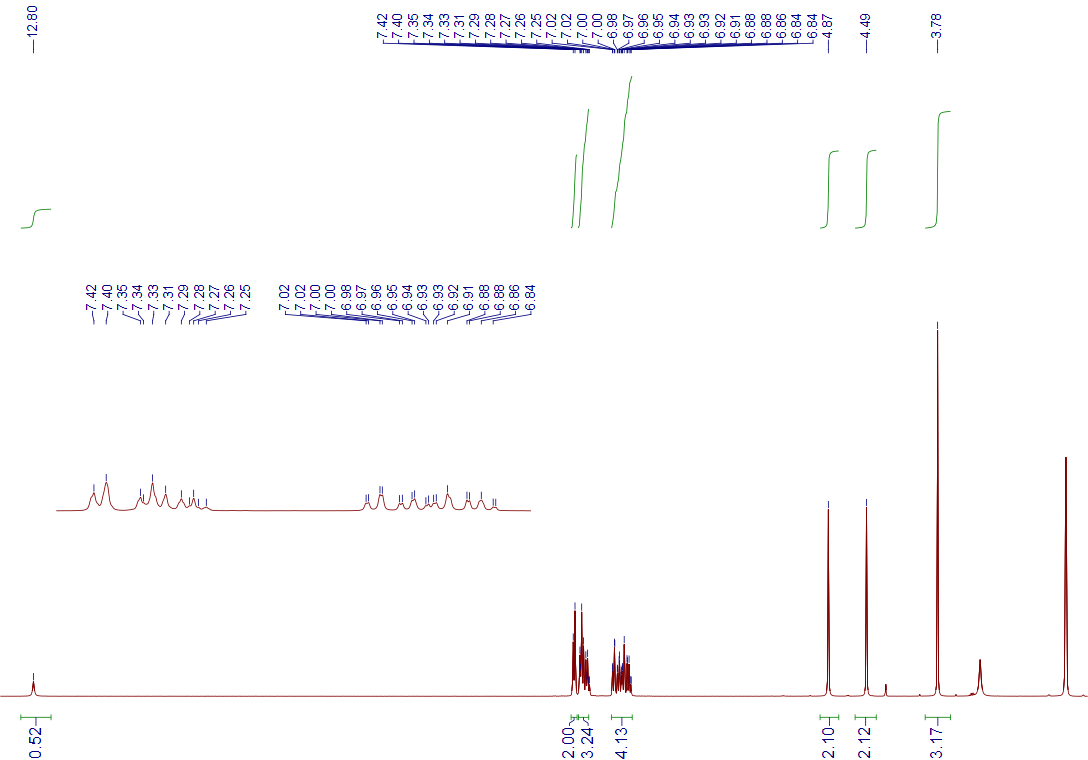


^1^H NMR of compound **5m**


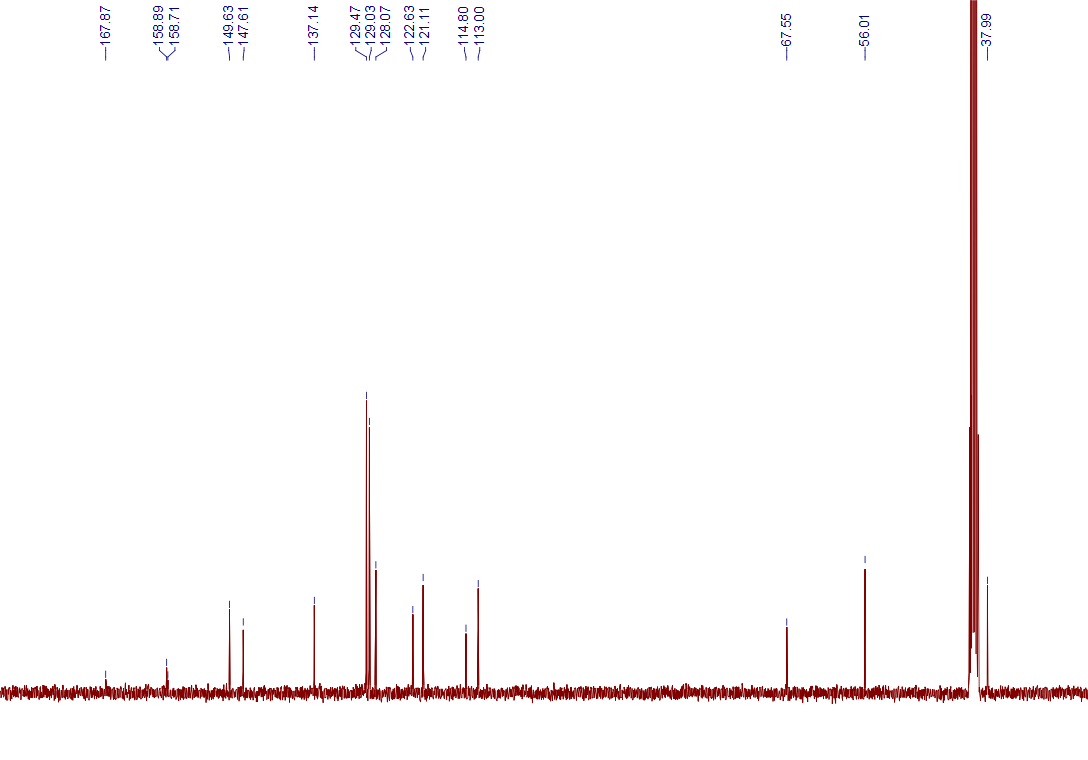


^15^C NMR of compound **5m**


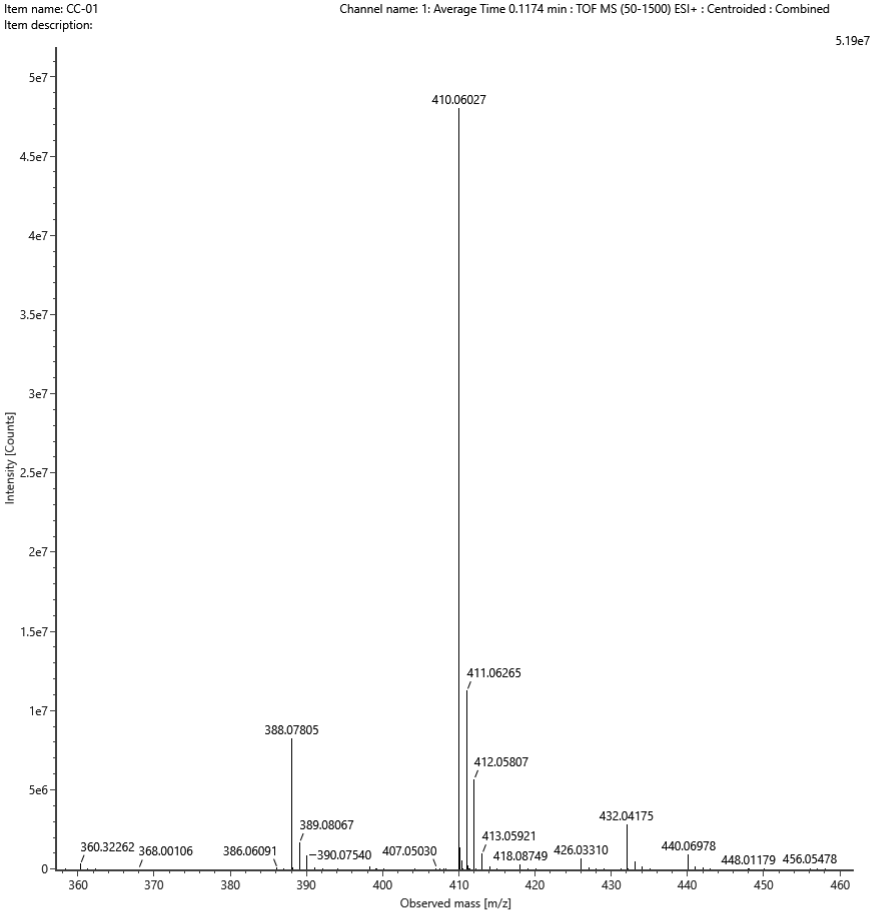


HRMS of compound **5m**


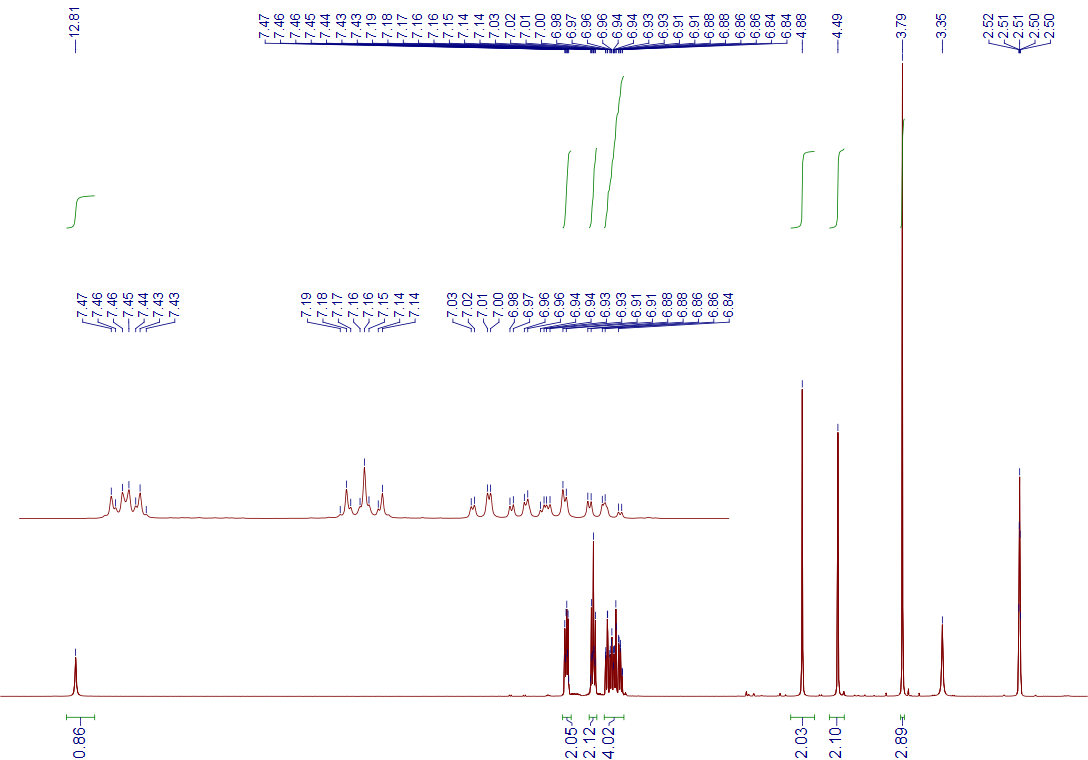


^1^H NMR of compound **5n**


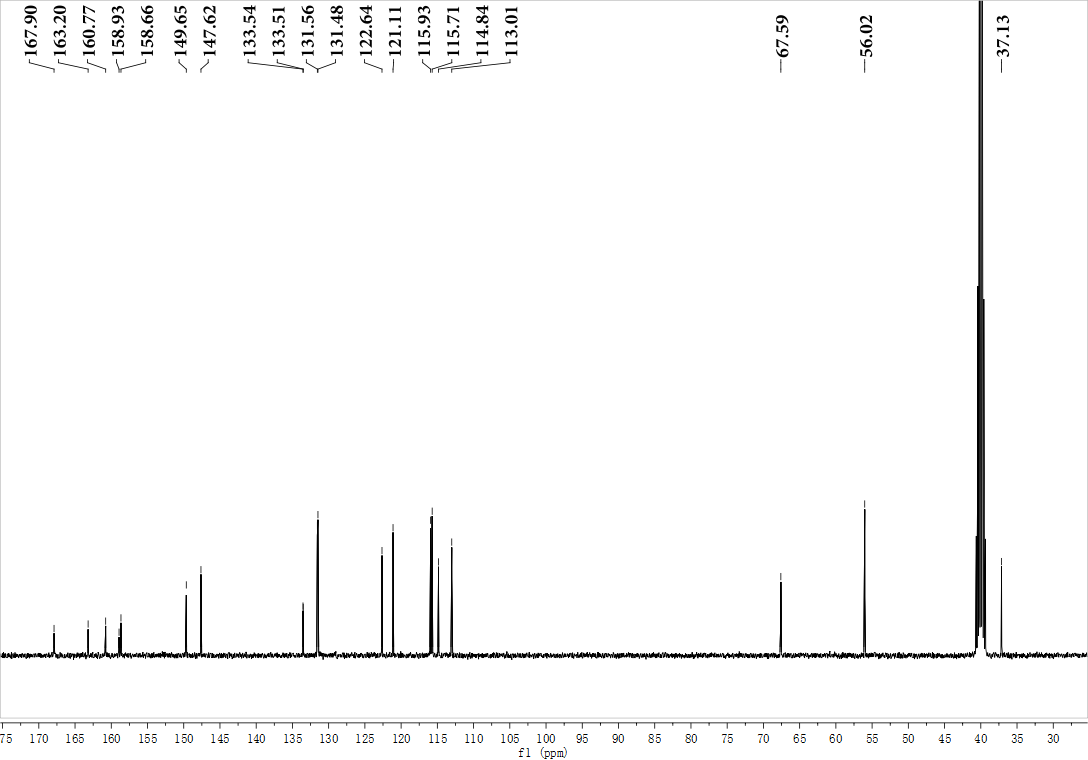


^15^C NMR of compound **5n**


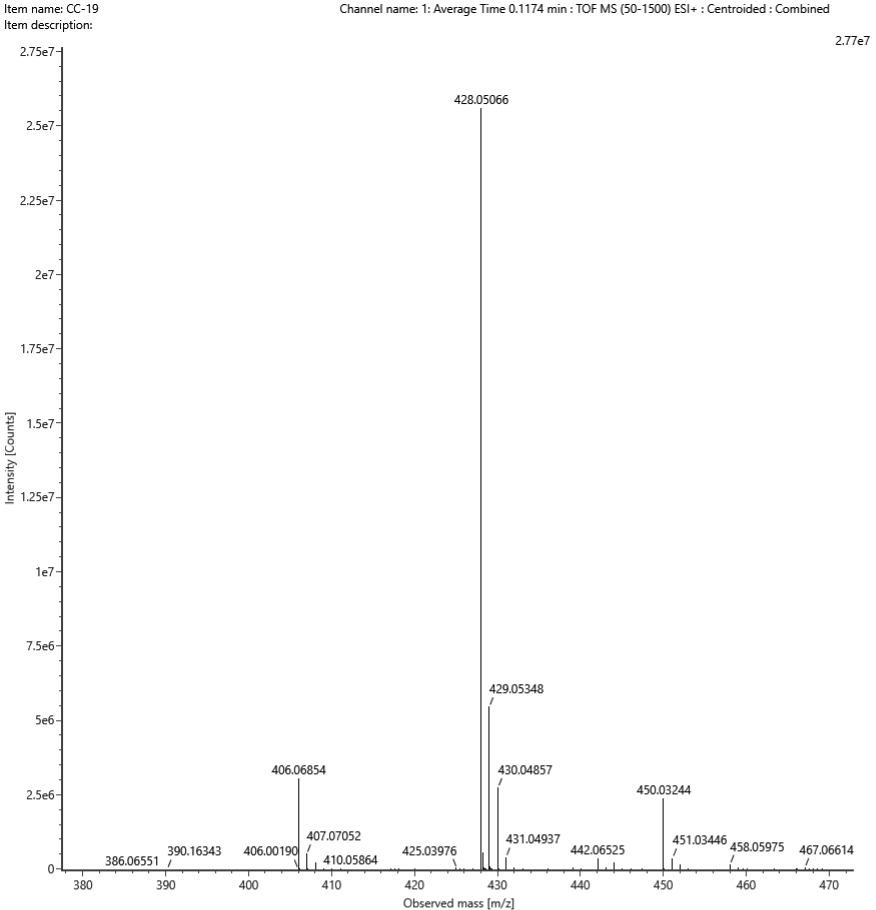


HRMS of compound **5n**


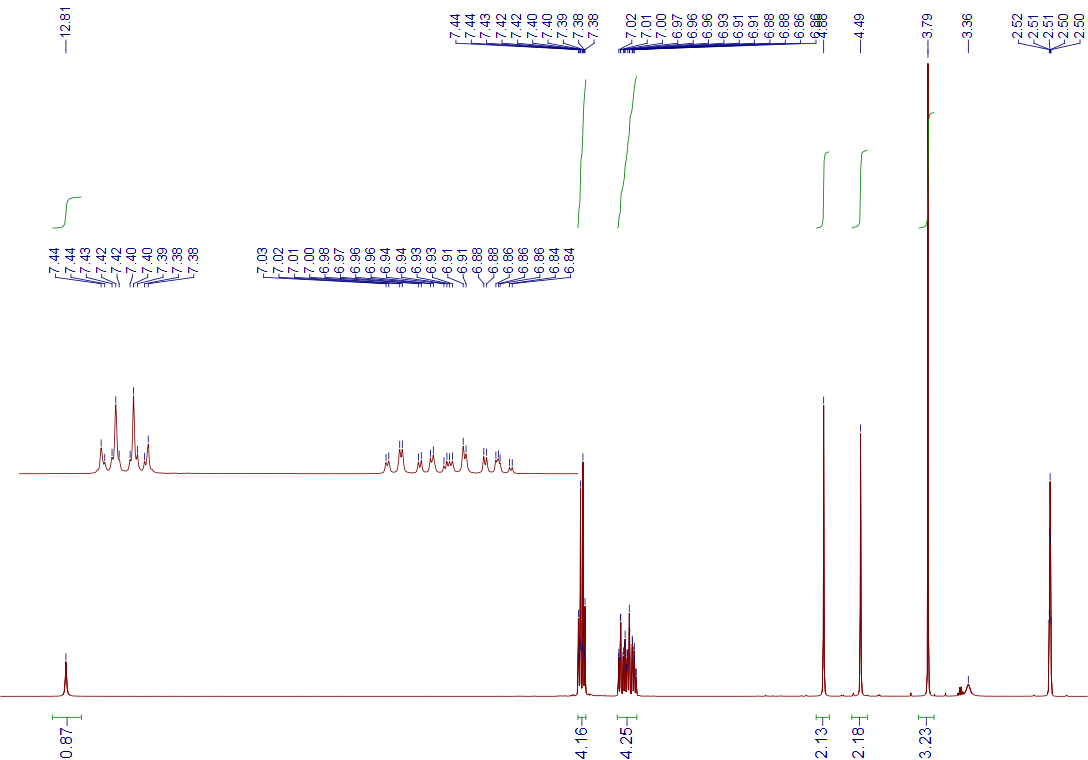


^1^H NMR of compound **5o**


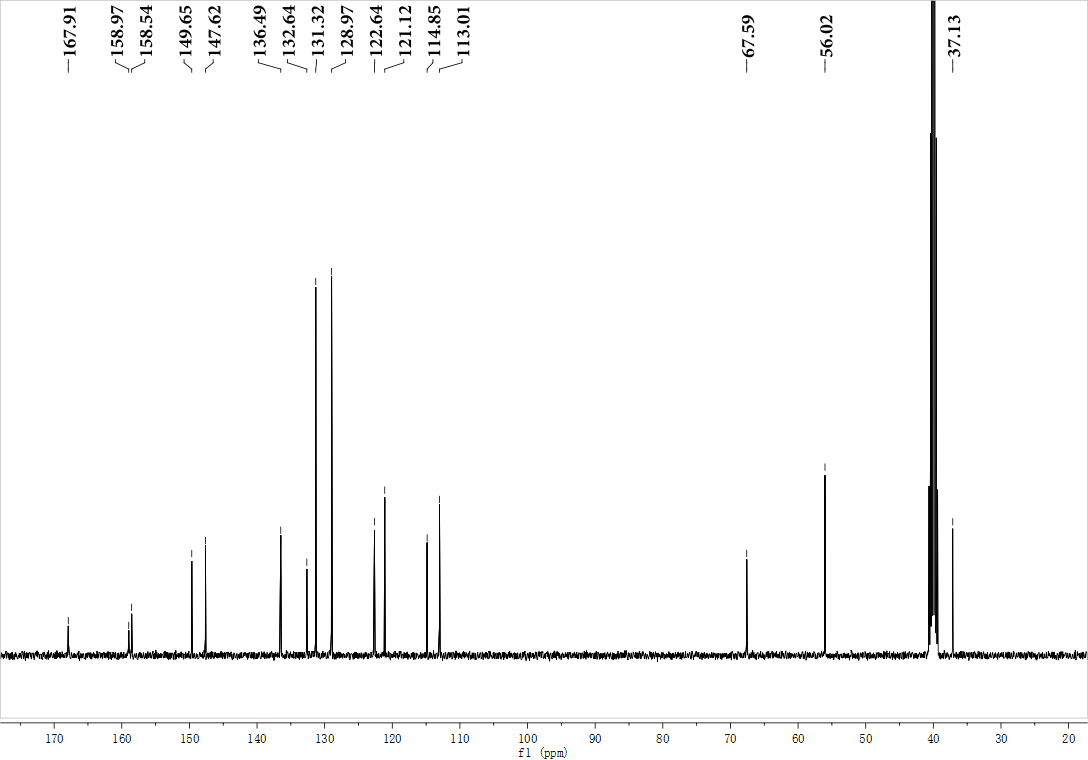


^15^C NMR of compound **5o**


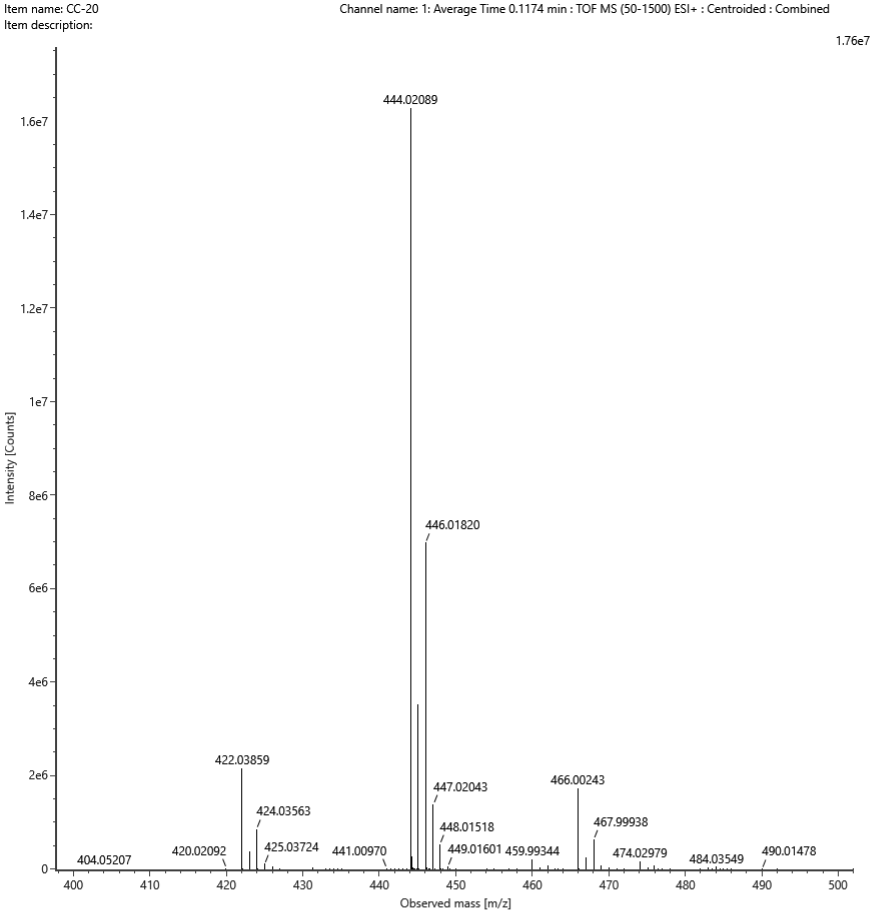


HRMS of compound **5o**


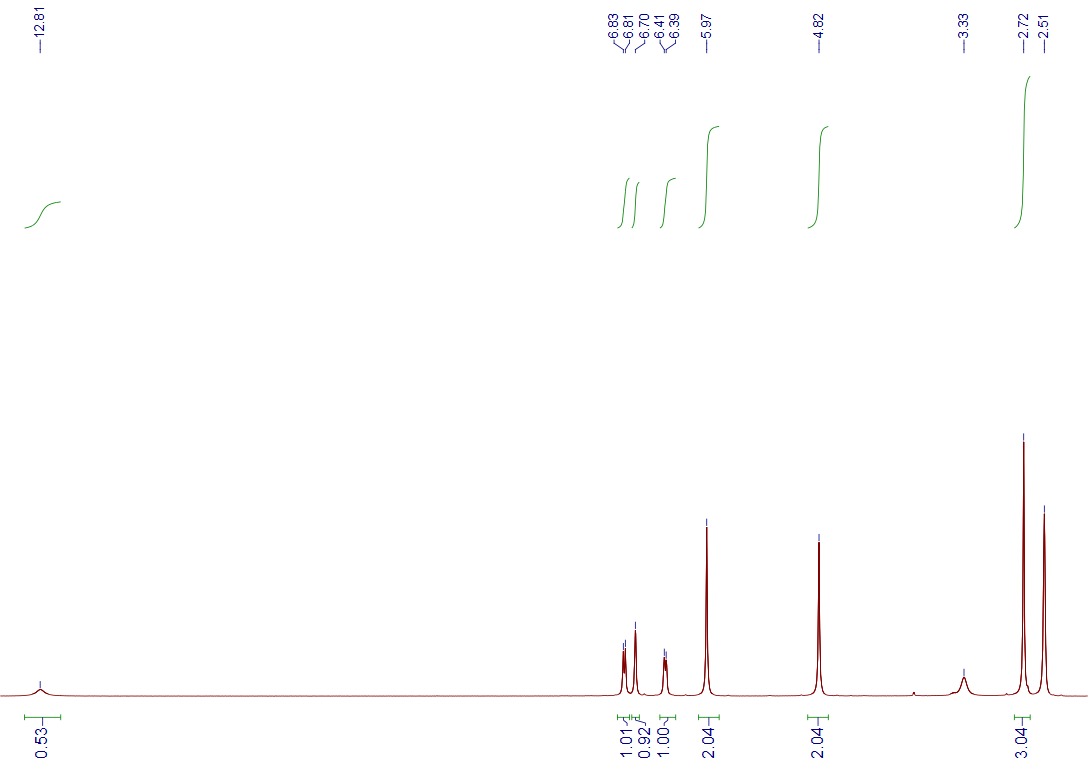


^1^H NMR of compound **5p**


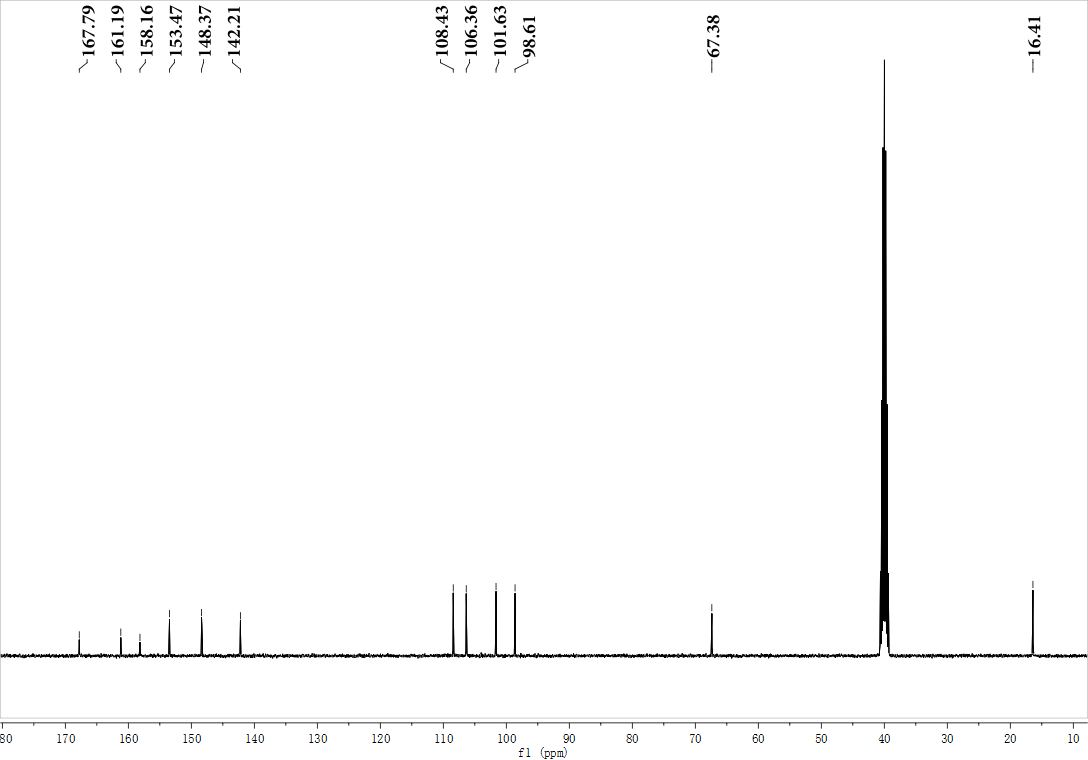


^15^C NMR of compound **5p**


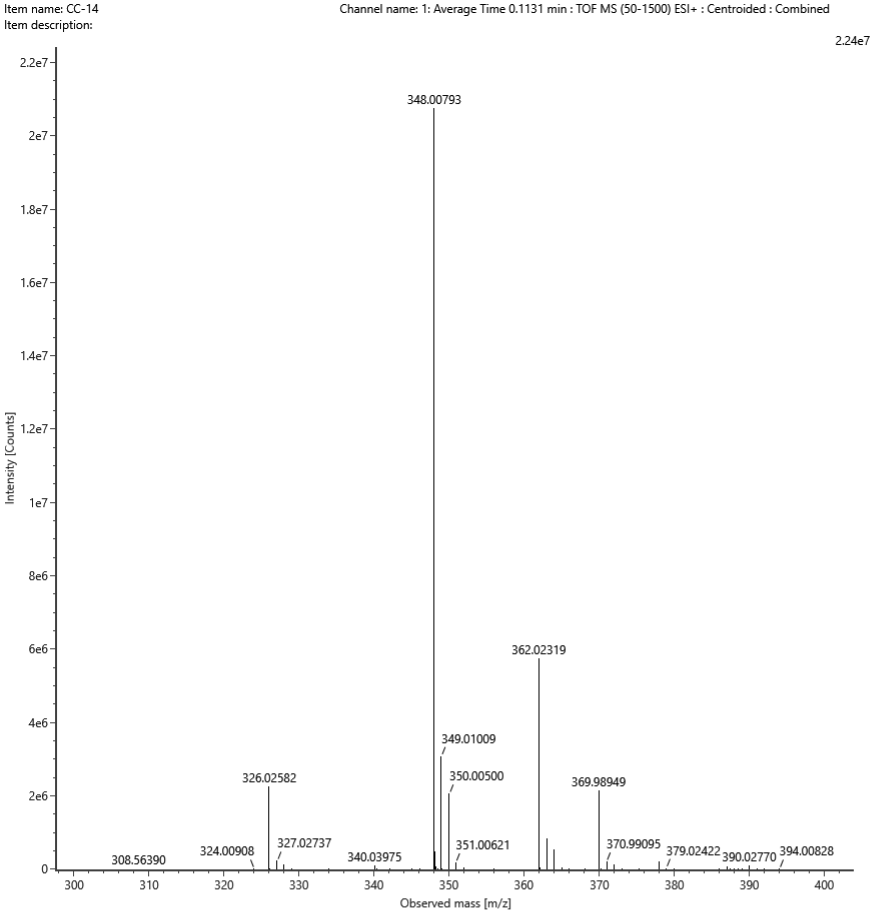


HRMS of compound **5p**


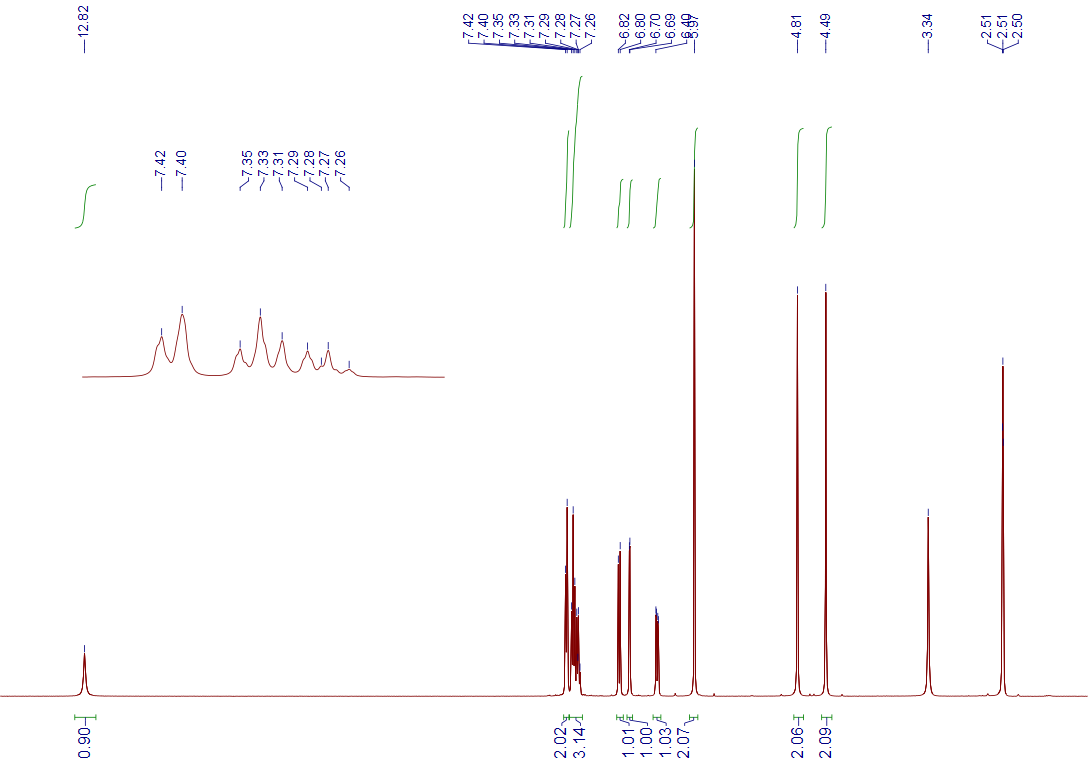


^1^H NMR of compound **5q**


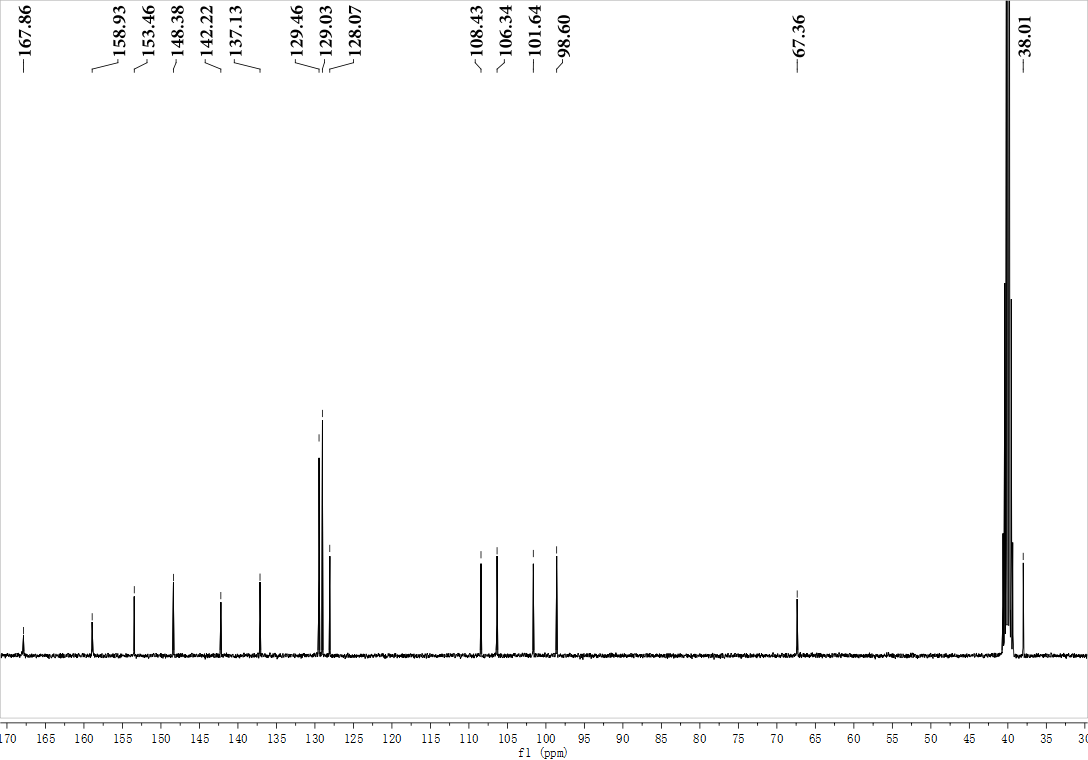


^15^C NMR of compound **5q**


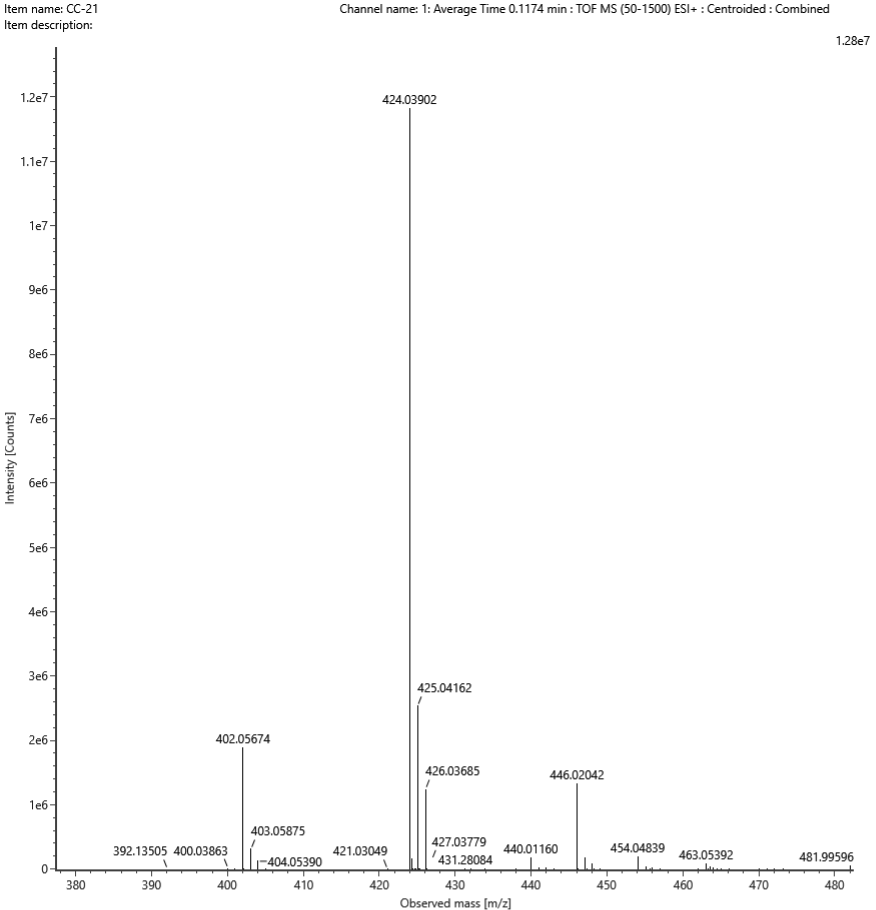


HRMS of compound **5q**


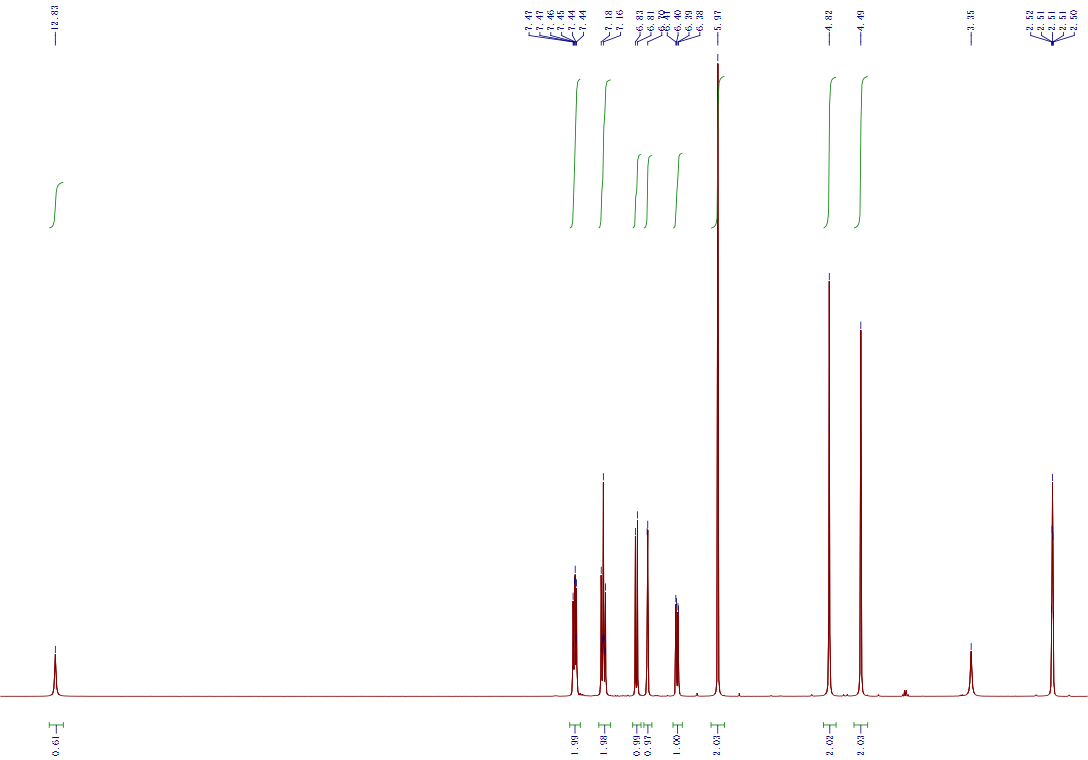


^1^H NMR of compound **5r**


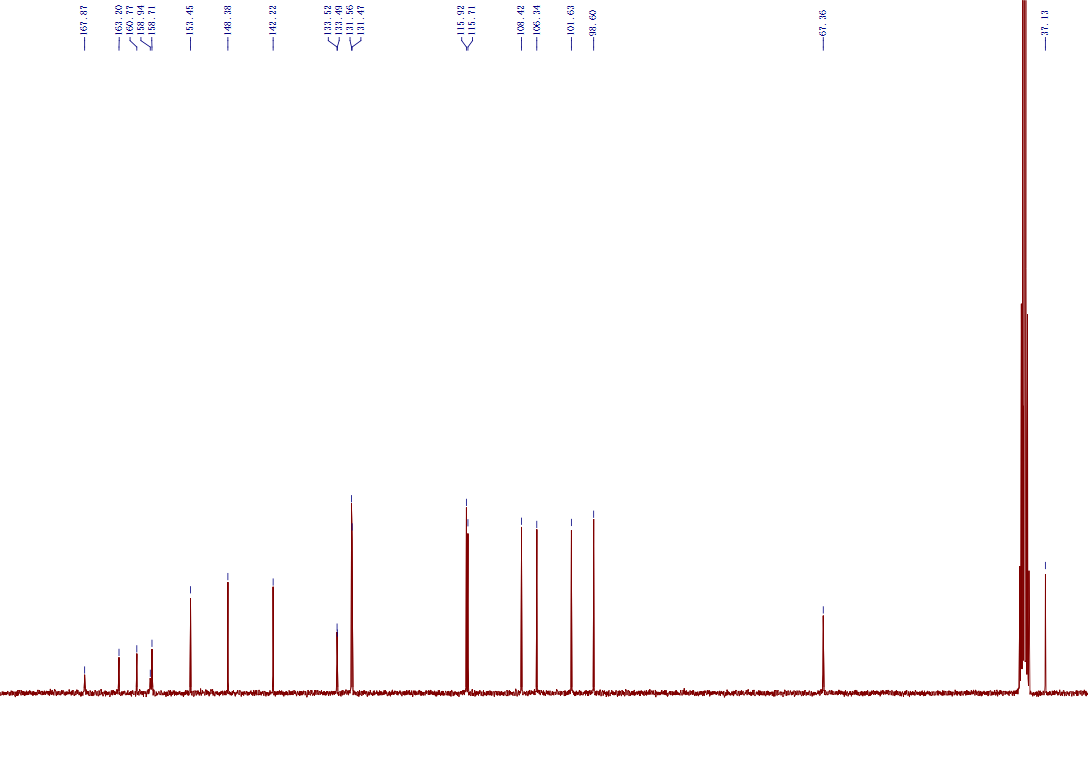


^15^C NMR of compound **5r**


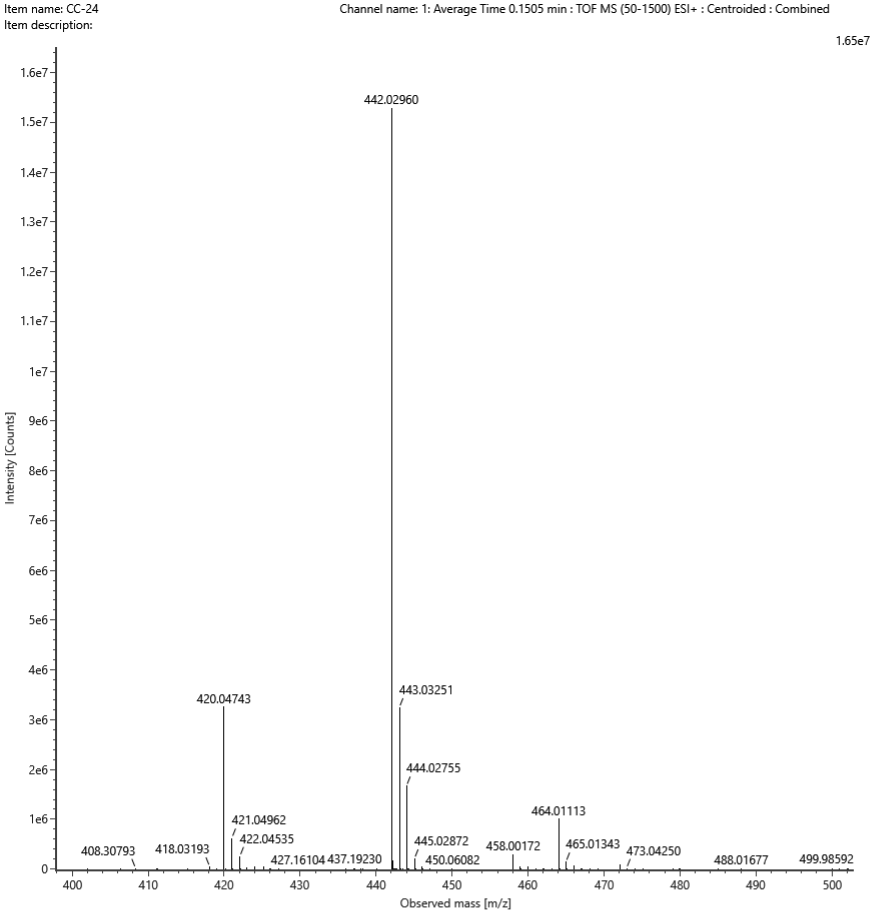


HRMS of compound **5r**


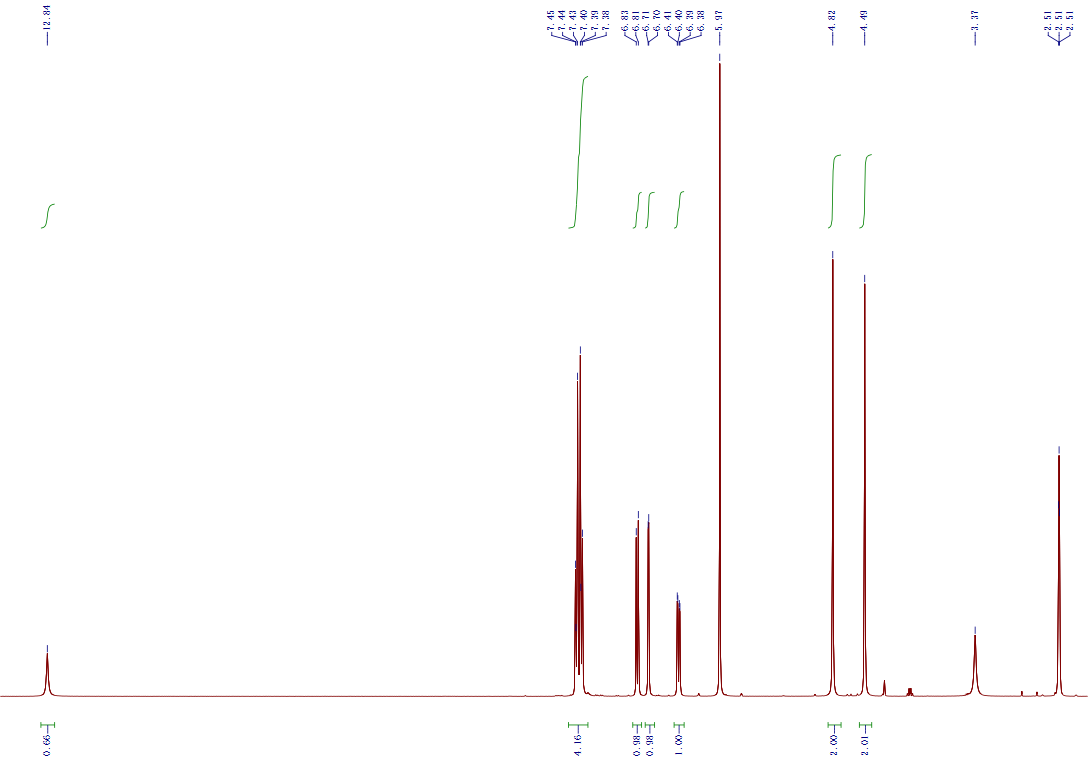


^1^H NMR of compound **5s**


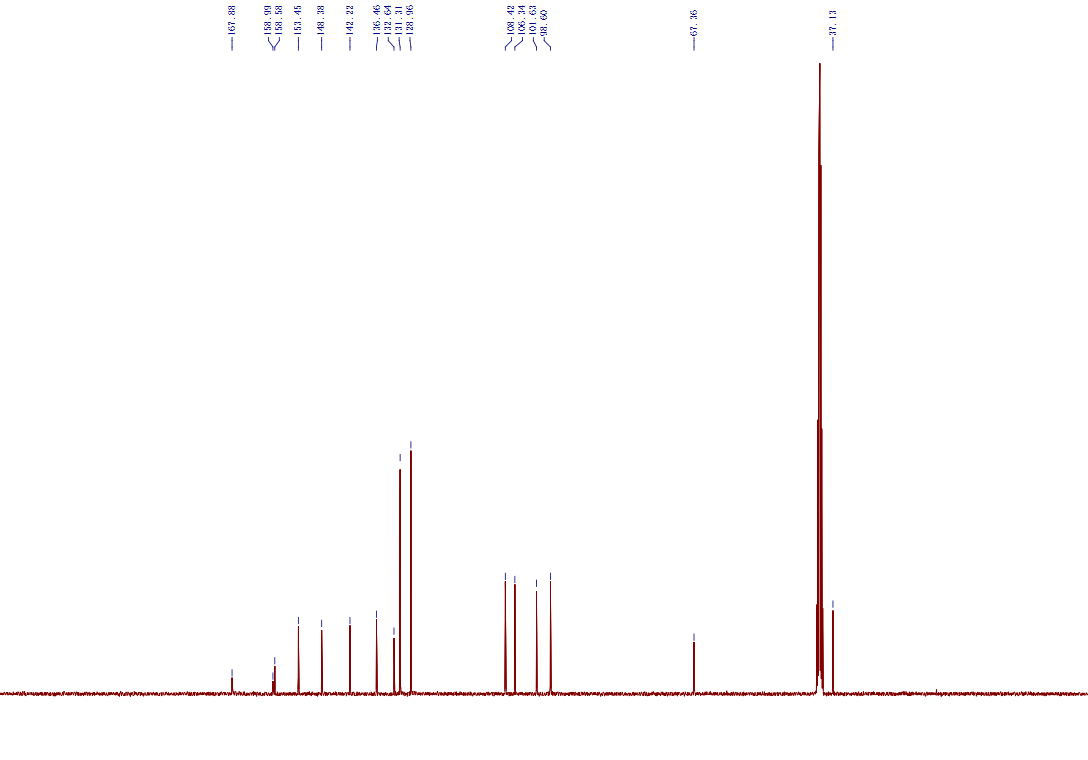


^15^C NMR of compound **5s**


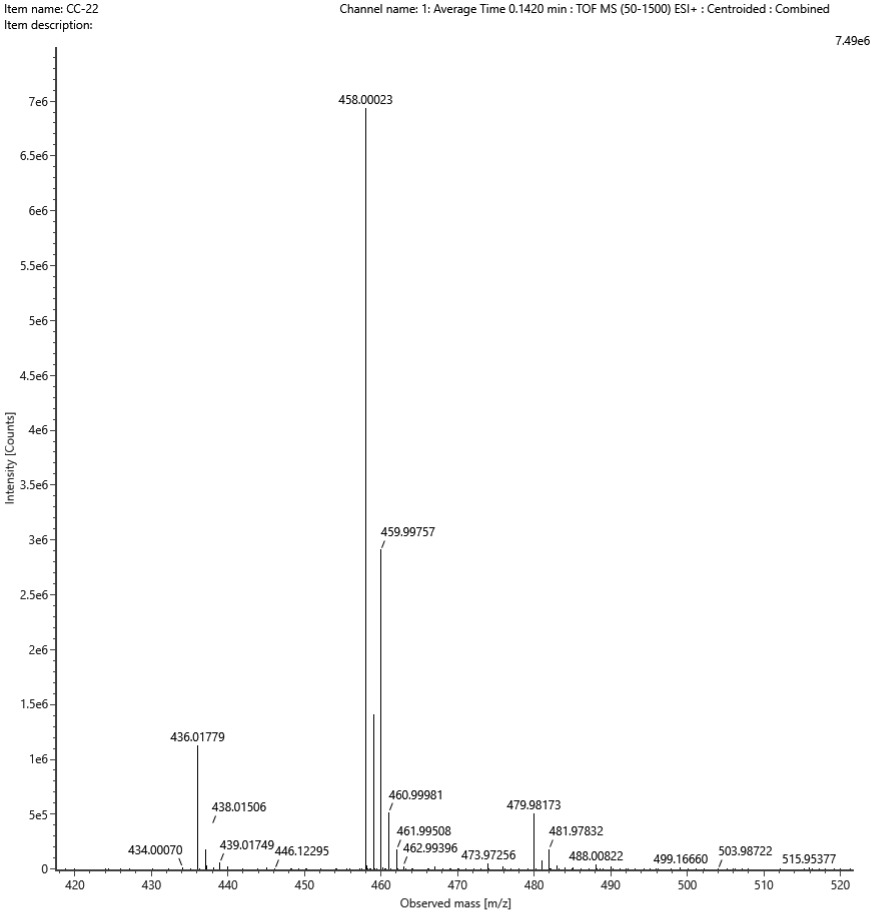


HRMS of compound **5s**
